# Low expression of *ALOX15B* modulates immunosuppressive tumor microenvironment in diffuse large B-cell lymphoma via the TAP1/MHC-I axis

**DOI:** 10.1186/s13046-025-03613-2

**Published:** 2026-01-12

**Authors:** Li Wang, Jiaying Liu, Yucui Shang, Lanxin Zhang, Muchen Zhang, Di Fu, Shu Cheng, Pengpeng Xu, Eurydice Anegeli, Guilhem Bousquet, Ying Fang, Yu Liu, Wei-Li Zhao

**Affiliations:** 1 State Key Laboratory of Medical Genomics; National Research Center for Translational Medicine at Shanghai, Shanghai Institute of Hematology, Ruijin Hospital Affiliated to Shanghai Jiao Tong University School of Medicine, No. 197 Ruijin Second Road, Shanghai, China; 2 Department of Hematology and Institute of Hematology, State Key Laboratory of Biotherapy and Cancer Center, West China Hospital, Sichuan University, No.17 People’s South Road, Chengdu, Sichuan China; 3 Assistance Publique Hôpitaux de Paris, Hôpital Avicenne, Service d’Oncologie Médicale, 93000 Bobigny, France; 4 Laboratory of Molecular Pathology, Pôle de Recherches Sino-Français en Science du Vivant Et Génomique, Shanghai, China

**Keywords:** ALOX15B, 17p deletion, Diffuse large B-cell lymphoma, Immunosuppressive tumor microenvironment, TAP1/MHC-I axis

## Abstract

**Background:**

Diffuse large B-cell lymphoma (DLBCL) patients with 17p deletion (17p^-^) show variable outcomes under R-CHOP (rituximab, cyclophosphamide, doxorubicin, vincristine, and prednisone) therapy. *ALOX15B* (arachidonate 15-lipoxygenase type B), located on chromosome 17p, regulates immune responses via arachidonic acid (AA) metabolism. This study investigates its role in DLBCL progression and explores its epigenetic regulation and therapeutic potential.

**Methods:**

We analyzed bulk and single-cell transcriptomic data from DLBCL cohorts to evaluate *ALOX15B* expression and its correlation with clinical outcomes, immune microenvironment, and therapy resistance. Functional assays using siRNA knockdown, luciferase reporter, and drug sensitivity experiments were performed in DLBCL cell lines. Murine and patient-derived xenograft (PDX) models were employed to assess tumor behavior and treatment efficacy in vivo. Chromatin immunoprecipitation sequencing (ChIP-seq), assay for transposase-accessible chromatin using sequencing (ATAC-seq), were conducted to explore the epigenetic regulation of *ALOX15B*.

**Results:**

Low *ALOX15B* expression was associated with inferior progression-free survival (PFS), immunosuppressive microenvironment, and reduced CD8 ^+^ T cell cytotoxicity in DLBCL. Mechanistically, *ALOX15B* deficiency led to upregulation of COX-2/PGE2 signaling and downregulation of the TAP1/MHC-I antigen presentation axis. Silencing *ALOX15B* promoted tumor cell proliferation and resistance to doxorubicin. Epigenetically, HDAC1/2 were enriched at the *ALOX15B* promoter region, repressing its expression. Treatment with the HDAC inhibitor tucidinostat restored *ALOX15B* expression, enhanced tumor cell apoptosis, reinstated antigen presentation, and reprogrammed the tumor immune landscape in both cell lines and in vivo models.

**Conclusions:**

*ALOX15B* is a key epigenetically regulated gene in DLBCL that modulates the tumor immune microenvironment and response to chemotherapy. Its downregulation promotes immune evasion and treatment resistance, while tucidinostat effectively restores its expression and anti-tumor immunity. These findings highlight *ALOX15B* as a prognostic biomarker and therapeutic target, particularly in 17p^−^ DLBCL.

**Supplementary Information:**

The online version contains supplementary material available at 10.1186/s13046-025-03613-2.

## Background

Diffuse large B-cell lymphoma (DLBCL) is the most common subtype of non-Hodgkin’s lymphoma [1], for which the combination of rituximab, cyclophosphamide, doxorubicin, vincristine, and prednisone (R-CHOP) remains the standard first-line treatment [2]. The prognosis for DLBCL patients with specific high-risk genetic abnormalities, such as chromosome 17p deletion (17p^−^), needs further investigation [3]. Although 17p^−^ has been linked to poor outcomes in various malignancies [4–6], its prognostic significance in DLBCL, especially in the context of R-CHOP immunochemotherapy, is inconclusive. Some studies suggest that patients with 17p^−^ exhibit poorer treatment responses and shorter progression-free survival (PFS), whereas others have found no significant differences in disease outcome, as compared to those without 17p^−^ deletions. This ongoing debate underscores the need for systematic and comprehensive investigations to better define the impact of 17p^−^ on treatment efficacy and patient survival. Understanding the underlying molecular mechanisms in these patients is critical for refining risk stratification and improving personalized treatment approaches in DLBCL.

Given these uncertainties, we aimed to explore the role of *ALOX15B*, a gene located on chromosome 17p, in the pathogenesis of DLBCL and its potential influence on treatment outcomes. *ALOX15B* plays a key role in the metabolism of arachidonic acid, producing lipid mediators, including prostaglandins, which are central to inflammation and immune regulation [7].

In this study, we first explored the pivotal role of *ALOX15B* in suppressing the tumor microenvironment in DLBCL in a histone acetylation-dependent manner. Then, we investigated the underlying mechanism of histone deacetylase inhibitor (HDACi) on *ALOX15B* regulatory circuitry and anti-tumor immunity in 17p^−^ DLBCL. Our findings provide both in vitro and in vivo evidence that *ALOX15B* functions as a novel epigenetic therapeutic target, offering promising implications for treating 17p^−^ DLBCL.

## Methods

### Study design

The flowchart in Supplementary Figure S1 describes the selection methods for patients with DLBCL. This study was based on transcriptomic data obtained from the tumor tissues of 360 patients with newly diagnosed DLBCL, sourced from two previously published research cohorts: 329 patients from a standard R-CHOP cohort [8] and 31 patients from a phase II study of tucidinostat plus R-CHOP in elderly patients with newly diagnosed DLBCL (NCT02753647) [9]. Histological diagnosis was established according to the World Health Organization classification [10] excluding mediastinal large B-cell lymphoma or primary central nervous system DLBCL. The treatment response was evaluated according to the International Workshop Criteria [11]. The Hospital Review Board approved the study, and informed consent was obtained following the Declaration of Helsinki.

### RNA sequencing

Total RNA from frozen tumor tissue or cells was extracted using TRIzol reagent (Thermo Fisher Scientific, MA, USA) and a RNeasy Mini Kit (Thermo Fisher Scientific, MA, USA). RNA sequencing libraries were constructed using a TruSeq RNA Samples Preparation Kit for Illumina. The concentration and size distribution of the RNA sequencing libraries were confirmed using Qubit (Thermo Fisher Scientific, MA, USA) and Agilent 2100 (Agilent). The read pairs were aligned to the reference RefSeq hg19 by Burrows-Wheeler aligner version 0.7.13-r1126. HTSeq was used to generate transcript count table files [12]. Bioinformatic analyses were performed using R 3.5.1 with the R package “sva.” Raw reads were normalized and differentially expressed genes were identified using the “limma” R package (v3⋅38⋅3). The heatmap was generated using Java TreeView [13]. Gene set enrichment analysis (GSEA) was performed to identify regulated pathways and determine global gene expression patterns [14]. Single-sample GSEA (ssGSEA) was used to calculate the enrichment scores of specific gene sets [15].

### Molecular classification

All tumor samples were categorized as germinal-center B-cell-like (GCB) and non-GCB subtypes based on the Hans algorithm, with a 30% cutoff value for CD10, BCL-6, and MUM1 [16]. For BCL2/MYC double expression (DE), the cutoff values of BCL2 and MYC were 50% and 40%, respectively [17].

### Cell lines

B-lymphoma cell lines DB (obtained from American Type Culture Collection, Manassas, VA, USA) and U2932 (Deutsche Sammlung von Mikroorganismen und Zellkulturen, Braunschweig, Germany) were grown in RPMI-1640 medium (MA0548, MeilunBio) with 10% heat-inactivated fetal bovine serum (FBS) (A5669701, Gibco) in a humidified atmosphere containing 95% air-5% CO_2_ at 37°C.

### Retroviral construction and small interfering RNAs

The MSCV-mirE-SV40-Myc-IRES-GFP retroviral vector was used to insert shRNA expression cassettes targeting *Alox15b* into the miR-E scaffold. Retroviral particle packaging and subsequent cell infection were performed as previously described [18]. Small interfering RNAs **(**siRNAs) involved in the study were constructed by Shanghai Xitu Biotechnology Co., Ltd. The siRNA sequences (5′−3′) were as follows: forward primer: GGAACAUGAAGCAGCUGAAtt; reverse primer: UUCAGCUGCUUCAUGUUCCtt.

### Luciferase-reporter assay

The promoter region (− 2000/+ 100) of the *TAP1* gene was PCR-amplified from human genome DNA. The primer sequences were as follows: forward primer: 5’- CCTGAGCTCGCTAGCCTCGAGAGTAACAGGCCAGAGCTGACTG −3’; reverse primer: 5’- CAGTACCGGATTGCCAAGCTTAATCTCGGGAGCAGCCCTGGTG −3’. The amplified 2.1kb fragment was then cloned into a pGL4.14 vector between XhoI and HindIII sites. The resulting plasmid was confirmed by Sanger sequencing. Luciferase reporter assay was conducted using Duo-Lite Luciferase Assay System (DD1205-01, Vazyme, Nanjing, China) according to the manufacturer’s protocols.

### Tumor dissociation and single-cell RNA sequencing (scRNA-seq)

Thirteen tumor samples were collected from 13 patients with newly diagnosed DLBCL. Frozen tumor samples were immediately thawed and washed with phosphate-buffered saline (PBS). Dead Cell Removal Kit (Miltenyi Biotec) was used to remove dead cells. The preparation of single-cell suspensions and the synthesis of complementary DNA and gene expression libraries were performed according to the manufacturer’s instructions. The chromium single cell 3’Kit v2 (10 × Genomics) was used for two samples, whereas the chromium single cell 5’Kit v2 (10 × Genomics) was used for 11 samples. The 3’ gene expression libraries were sequenced on a Novaseq 6000 (Illumina), whereas the 5’ gene expression libraries were sequenced on a MGI-2000 sequencer. scRNA-seq FASTQ files were preprocessed using Cell Ranger v6.0.1 (10 × Genomics) with the default parameters.

### Processing of scRNA-seq data

Downstream analysis was performed using R (v4.4.1). The Seurat package (v4.4.0) was used for quality control, normalization, dimensionality reduction, and clustering. Genes detected in less than 3 cells were filtered out. To ensure quality, cells with fewer than 200 unique molecular identifiers (UMIs) or 200 detected genes were excluded, along with potential doublets identified as cells containing more than 15,000 UMIs or 6000 genes. Mitochondrial contents above 15% were also excluded. The filtered matrix was log-normalized using the NormalizeData function. To mitigate the effects of cell cycle heterogeneity on cell clustering, the CellCycleScoring function was applied to each cell for scoring the S and G2M phases. Variable genes were identified using the FindVariableFeatures function with the ‘vst’ method. Ribosome- and mitochondria-related genes were removed from highly variable genes. The features were then centered and scaled using the ScaleData function, regressing out the effect of mitochondrial gene content and scoring of the S and G2M phases.

### Dimensionality reduction and clustering

Dimension reduction was conducted via principal component analysis (PCA) using the RunPCA function. To eliminate batch effects between datasets, the RunHarmony function in Harmony package (v1.2.0) was applied to the merged datasets [19]. The K‐nearest neighbor for the dataset was computed using the FindNeighbors function. Clustering was performed using the FindClusters function with the Louvain algorithm. The RunUMAP function was used to visualize cells in two-dimensional embeddings. The major cell type was identified based on classical marker genes reported in the literature. Doublets were identified by expressing two or more sets of well‐studied canonical markers of major cell types and were excluded [20].

### Identification of malignant B cells

Due to the inter-patient tumor heterogeneity, malignant cells tend to show a distinct clustered distribution compared to normal cells. In other words, non-malignant cells from different patients usually cluster together by cell type, whereas malignant cells from different patients usually form separate clusters [19]. Thus, B cell subpopulations were extracted and split by sample in this study. After removing the doublets from the B cells of each sample, the B cells were merged without the elimination of batch effects. Clusters B2 and B20 consisted of multiple samples and were considered normal B cells. Then, InferCNV package (v1.20.0) [21] was used to estimate the chromosomal copy number variation (CNV) with cluster B11 as a reference, before verifying the above results.

### Differential expression and enrichment analysis

The FindAllMarkers and FindMarkers functions in Seurat were used to identify differentially expressed genes (DEGs). DEGs with an average log2 (fold change) > 0.5 and *P*‐values < 0.05 were included for KEGG enrichment and analyzed by clusterProfiler package (v4.12.6). DEGs with an average log2 (fold change) > 0.01, those with *P*‐values < 0.05, and those that were expressed in at least 25% of cells in at least one subgroup were used for GSEA. C2 (Reactome) genesets from MSigDB via the msigdbr package (v7.5.1) were used as a geneset for enrichment by the fGSEA package (v1.30.0).

### Cell–cell interaction

The Cellchat package (v2.1.2) [22] was used to analyze intercellular communication. The overall information flow of each signaling pathway was compared between the *ALOX15B* high and low groups using the rankNet function.

### Chromatin immunoprecipitation sequencing (ChIP-seq)

Nuclear extracts were derived from a total of 2 × 10^7^ cells per sample. Immunoprecipitation was performed using rabbit anti-human HDAC1 (ab280198, Abcam, Cambridge, MA, USA), HDAC2 (ab124974, Abcam, Cambridge, MA, USA) and HDAC3 (ab137704, Abcam, Cambridge, MA, USA) antibodies, with normal IgG (3900, Cell Signaling Technologies) used as a negative control. Subsequently, the DNA libraries underwent 15 cycles of amplification and were subjected to sequencing using the Illumina NovaSeq 6000 platform with paired-end 2 × 150 sequencing mode. To ensure the acquisition of high-quality clean reads, raw reads underwent a filtration process that involved the removal of sequencing adapters, short reads (length < 35 bp), and low-quality reads using Cutadapt (v1.9.1) [23] and Trimmomatic (v0.35) [24]. FastQC was then used to ensure high read quality. Peak detection was performed using the MACS (v2.1.1) [25] peak finding algorithm, with the *p*-value cutoff set as 0.05. Annotation of peak sites to gene features was performed using the ChIPseeker R package [26].

### Immunohistochemistry

Immunohistochemistry was performed on 5-μm-paraffin sections using an indirect immunoperoxidase method using antibodies against HDAC1 (A19571, ABclonal, Wuhan, China), HDAC2 (ab32117, Abcam, Cambridge, MA, USA) and ALOX15B (MA5-25,853, Invitrogen, Carlsbad, CA, USA). Expression levels were assessed according to the percentage of positive cells: + denoted < 25%; + + denoted 25%–49%; + + + denoted 50%–74%; + + + + denoted 75%–100%.

### Patient-derived tumor xenograft model

Four-week-old female NSG and NOD-SCID mice were obtained from Shanghai Laboratory Animal Center (Shanghai, China) for the establishment and maintenance of PDX models. Heterotopic PDX models were generated as previously described [27]. Decitabine and doxorubicin, either alone or in combination, were applied to PDX models with a low passage number (P3-P5) to preserve the genetic integrity of the parental tumors. Tumor volumes were calculated as 0.5 × a × b^2^, where “a” is the length and “b” is the width. Treatments were started after the tumor reached approximately 0.5 cm × 0.5 cm on the surface (day 0). The dose and administration schedule were as follows: doxorubicin 0.6 mg/kg twice a week, and tucidinostat 12.5 mg/kg/day for 2 weeks, while the control group was treated with a vehicle solution consisting of 0.2% carboxymethylcellulose saline and 0.1% Tween 80, as previously described [27]. Tumor-bearing mice were then euthanized by CO_2_ asphyxiation. Animals were used according to the ARRIVE guidelines and the protocols approved by the Shanghai Rui Jin Hospital Animal Care and Use Committee.

### Tumor models of immunocompetent mice

C57BL/6 mice (Taconic; 6–8-week-old, female) were obtained from the Institutional Animal Care and Use Committees of Sichuan University (Sichuan, China). The mouse lymphoma development experiments were conducted as previously described [18]. Bone marrow-derived B220 + B progenitor cells were transduced with retroviruses (described above) and then administered via tail-vein injection into C57BL/6 mice that had been sublethally irradiated (4.5 Gy, Cs137). All mice were randomly grouped before transplantation and were monitored twice a week until the start of treatment. Drug treatment was initiated 7 days after transplantation to ensure tumor engraftment in the sh*Alox15b* group. The mice were randomly assigned to two groups: one group was treated with vehicle solution consisting of 0.2% carboxymethylcellulose saline and 0.1% Tween 80, while another group received tucidinostat at a dose of 12.5 mg/kg/day administered once daily via oral gavage. Pharmacological treatment was administered until tumors were observed in all subjects in the vehicle group. scRNA-seq analysis was performed on lymph nodes collected from the sh*Ren* and sh*Alox15b* (vehicle [*n* = 3] and tucidinostat [*n* = 3]) groups and washed with PBS supplemented with 2% FBS. The ground lymph node tissue was filtered through a 100-μm filter to obtain a single-cell suspension. The cell numbers were quantified, and the concentration was adjusted to 800–1200 cells/μl, with cell activity > 85%. Single-cell suspensions were subjected to scRNA-seq within an hour of acquisition, using the 10 × genomics platform to construct the single-cell library of samples.

scRNA-seq libraries were generated using Chromium Single Cell 3’Reagent Kits v3 (10 × Genomics). To identify tumor cells, we added the exogenous plasmid sequence into the mm10 genome reference. Cellranger (v7.0.1) was used to generate the index of the genome reference and align the raw data with the mm10 genome reference for the murine model. Cells with fewer than 200 detectable genes were regarded as poor-quality data for removal. Poor-quality genes detected in three cells were also removed for further analysis.

The Seurat (v4.3.0) pipeline was used for visualization. The FindVariableFeatures function was implemented to identify 4000 high-variable genes using the ‘vst’ model. Scalarization in the Seurat pipeline was performed to determine the expression of high-variable genes for dimension reduction, identifying 30 PCA components. tSNE was used, following the previous pipeline, with 20 PCA components to visualize the dimensional reduction map of the cell clusters. The major subpopulations were identified using classical signatures, and the top markers were calculated using FindAllMarkers.

CellChat (v2.1.2) was used to quantify intrinsic cell–cell interaction signaling between tumor cells and other immune cells. GSEA was utilized to identify significantly enriched pathways from the Molecular Signatures Database using default parameters for each cell subtype.

### Western blot

Western blot was conducted following the procedures described previously [PMID: 37798292]. Samples were isolated and lysed in a lysis buffer (200 μl) (Sigma Aldrich, Shanghai, China). Protein lysates (10 μg) were electrophoresed on 10% sodium dodecylsulfate-polyacrylamide gel electrophoresis (SDS-PAGE) and transferred to nitrocellulose membranes (BioRad, Hercules, CA, USA). Membranes were blocked for non-specific binding using 5% nonfat dried milk and left overnight at 4 °C rocking at low speed with ALOX15B (SAB2100110, Sigma-Aldrich, MUC, GER), TAP1 (11,114–1-AP, Proteintech, CHI, USA), HLA class I ABC (15,240–1-AP, Proteintech, CHI, USA), HLA-ABC (HA723415, HUABIO, Hangzhou, CHN), GAPDH (3683S, Cell Signaling Technology, Danvers, MA, USA), ACTIN (A00730-100, GenScript, NJ, USA) as primary antibodies. A horseradish peroxidase-conjugated antibody was employed for secondary detection. The immunocomplexes were visualized using an enhanced chemiluminescence detection kit.

### Quantitative real-time PCR

Total mRNA was isolated using TRIzol reagent (Invitrogen, Carlsbad, CA, USA). Complementary DNA was synthesized using HiScript III RT SuperMix for qPCR (+ gDNA wiper) (R323-01, Vazyme, Nanjing, China). Quantitative real-time PCR (qRT-PCR) was performed using ChamQ Universal SYBR qPCR Master Mix (Q711-03, Vazyme, Nanjing, China). For human samples, primers targeting *ALOX15B*, *TAP1*, *HLA-A*, *HLA-B*, *HLA-C*, and *GAPDH* (endogenous control) were used. For mouse samples, primers targeting *Tap1*, *H2-Q4*, *H2-Q6*, *H2-Q7*, and *Gapdh* (endogenous control) were used. Relative expressions were calculated by the method of _ΔΔ_CT. The primer sequences of the above genes are listed in Supplementary Table 1.

### Statistical analysis

The clinical characteristics of the enrolled patients with DLBCL were analyzed using Pearson’s χ2 test or Fisher’s exact test. Correlation analysis was performed using Spearman’s rank correlation method. PFS was measured from the date of disease diagnosis to the date of disease progression or the date of the last follow-up. OS was defined from the date of disease diagnosis to the date of death or last follow-up. The Kaplan–Meier method was used to estimate survival functions using the log-rank test. A two-tailed Student’s *t*-test was used to compare the normalized expression of related genes and the proportion of immune cells. Experimental data from three separate experiments are presented as the mean ± SD and were analyzed using a two-tailed Student’s *t*-test. Statistical analyses were performed using the Statistical Package for the Social Sciences (SPSS) 26.0 software (SPSS Inc., Chicago, IL) or the GraphPad Prism 6 program. *P*-values < 0.05 were considered statistically significant.

## Results

### Low *ALOX15B* expression is associated with inferior clinical outcomes in patients with DLBCL

To investigate the prognostic impact of 17p^−^ in patients with DLBCL, we analyzed the transcriptomic data of 329 patients who received standard R-CHOP treatment from a previously published cohort of 576 cases [8] as shown in Supplementary Figure S1. We assessed the expression of 195 genes (Supplementary Table S2) on 17p.13 and their prognostic impact. By integrating differential gene analysis and Kaplan–Meier survival analysis, we identified five genes whose downregulation was associated with inferior PFS in patients with DLBCL treated with R-CHOP, including *ALOX15B*, *TAX1BP3*, *MYH8*, *PITPNM3*, and *KIF1C* (Supplementary Figure S2A). Receiver operating characteristic (ROC) curve analysis revealed that *ALOX15B* had the most significant effect on predicting the PFS of patients (Supplementary Figure S2B). However, the expression of *ALOX15B* was independent of the 17p- status (Supplementary Figure S2C).

**Fig. 1:**
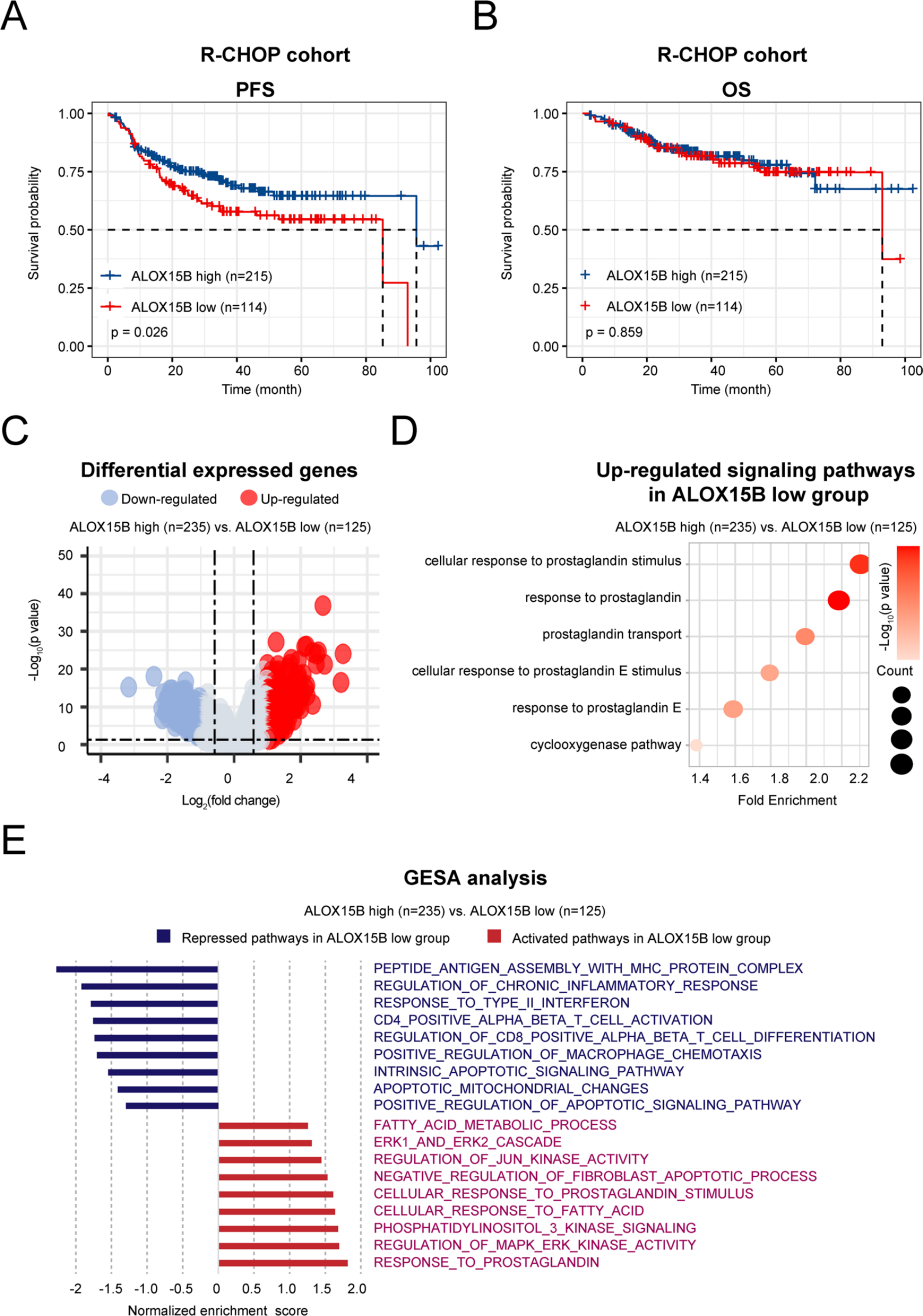
Survival analysis and molecular profiling of *ALOX15B* expression. **A**-**B** Progression-free survival (PFS) (**A**) and overall survival (OS) (**B**) of patients in the R-CHOP cohort categorized by high (*n* = 215) and low (*n* = 114) *ALOX15B* expression. **C** Volcano plot of differentially expressed genes between the *ALOX15B* high (*n* = 235) and low (*n* = 125) groups, with downregulated genes (blue) and upregulated genes (red) based on the log_2_ (fold change) and − log_10_ (*p*-value). **D** Upregulated signaling pathways in the *ALOX15B* low group. The size of the circle corresponds to the pathway enrichment count. **E** Gene set enrichment analysis (GSEA) of repressed and activated pathways in the *ALOX15B* low group compared to the high group

We then divided patients into *ALOX15B* high expression (*ALOX15B* high, *n* = 215) and *ALOX15B* low expression (*ALOX15B* low, *n* = 114) groups according to the ROC cutoff value of *ALOX15B,* expression values equal or greater than 1.539 were classified into high *ALOX15B* expression, whereas values less than 1.539 were considered low expression. As shown in Figs. 1A and B, patients in the *ALOX15B* low group (*n* = 114) exhibited inferior PFS than those in the *ALOX15B* high group (*n* = 215) treated with R-CHOP. However, no significant differences in the overall survival (OS) were observed between the two groups. The clinical and pathological characteristics of the patients are summarized in Table 1. The clinical characteristics did not differ significantly between patients with high and low *ALOX15B* expression.

**Table 1:** Clinical characteristics of 329 DLBCL patients in R-CHOP cohort

	*ALOX15B* high n = 215		*ALOX15B* low *n* = 114		
	n	%	n	%	*p* value
Age					
Median (range)	60 (19–85)		56 (17–83)		
≤ 60	114	53.0%	72	56.5%	0.078
> 60	101	47.0%	42	43.5%	
Sex					
Male	117	54.4%	69	60.5%	0.296
Female	98	45.6%	45	39.5%	
ECOG					
0–1	205	95.3%	105	92.1%	0.320
≥ 2	10	4.7%	9	7.9%	
Stage					
I/II	124	57.7%	73	64.0%	0.289
III/IV	91	42.3%	41	36.0%	
LDH					
Normal	106	49.3%	50	47.4%	0.347
Elevated	109	50.7%	64	52.6%	
Extranodal sites					
0–1	173	80.5%	90	78.9%	0.7732
≥ 2	42	19.5%	24	21.1%	
HANS					
GCB	84	39.1%	32	28.1%	0.0527
Non-GCB	131	60.9%	82	71.9%	
MYC/BCL2 double expressor					
Yes	47	21.9%	28	24.6%	0.5833
No	168	78.1%	86	75.4%	

### Low *ALOX15B* expression mediated COX-2/PGE2 upregulation and is associated with immunosuppressive tumor microenvironment within DLBCL

We collected tumor transcriptomic data (*n* = 360), stratified the samples into low (*n* = 125) and high (*n* = 235) *ALOX15B* expression groups, and performed differential gene expression analysis (Fig. 1C). Gene ontology (GO) enrichment analysis revealed significant enrichment of several prostaglandin-associated biological processes in the low *ALOX15B* expression group, consistent with previous findings (Fig. 1D). GSEA indicated a remarkable downregulation of immune-related pathways in tumors from patients with low *ALOX15B* expression, including processes such as antigen presentation complex assembly, inflammatory response, and interferon production, in addition to apoptosis pathways. In contrast, pathways associated with prostaglandin signaling, as well as various oncogenic pathways, were significantly upregulated in the low *ALOX15B* expression group (Fig. 1E). These results suggested that reduced *ALOX15B* expression may suppress tumor apoptosis and modulate immune processes by altering prostaglandin metabolism.

### Low *ALOX15B* expression is associated with reduced MHC-I–mediated antigen presentation in malignant B cells

**Fig. 2:**
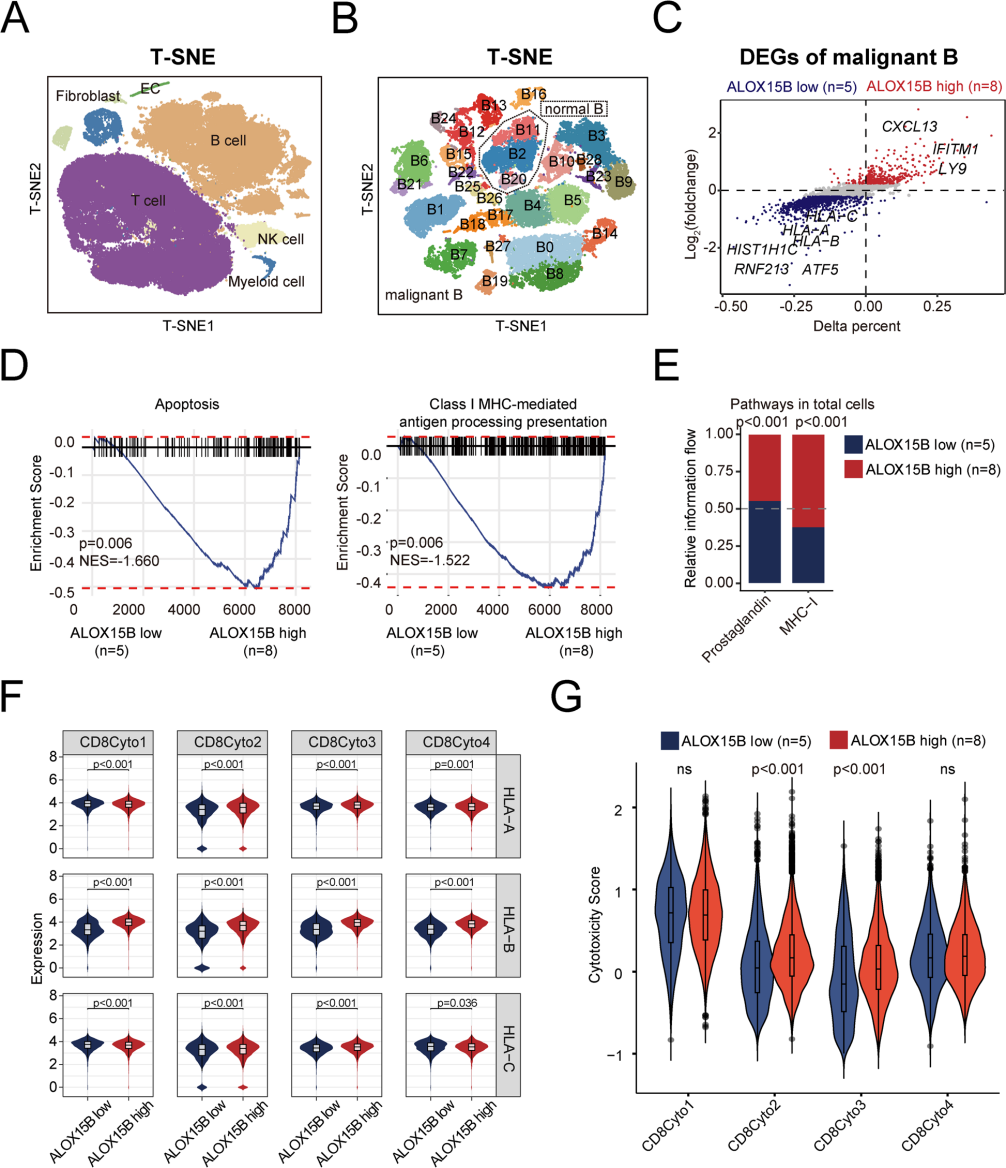
Single-cell analysis of *ALOX15B* expression in malignant B and T cells. **A** t-SNE plot showing the clustering of immune cell types, including fibroblasts, endothelial cells (ECs), B cells, T cells, natural killer (NK) cells, and myeloid cells, based on gene expression profiles. **B** t-SNE plot illustrating the clustering of normal and malignant B cell subsets. Normal B cells are in the dotted circle, and malignant B cells are outside of the circle. **C** Differentially expressed genes (DEGs) in malignant B cells from high and low *ALOX15B* expression groups. **D** GSEA of apoptosis and MHC-I-mediated antigen processing and presentation in the high and low *ALOX15B* expression groups. **E** Relative information flow in total cells showing a significant difference in the relative flow of information through the prostaglandin and MHC-I signaling pathways between the high and low *ALOX15B* expression groups (*p* < 0.001). **F** Expression of MHC-related molecules in CD8^+^T cell clusters between high and low *ALOX15B* expression groups. **G** Cytotoxicity scores in different CD8 ^+^ cytotoxic T cell subsets (CD8Cyto1, CD8Cyto2, CD8Cyto3, CD8Cyto4) between the high and low *ALOX15B* expression groups

We next conducted a single-cell transcriptomic analysis of tumor samples from 13 patients, aiming to understand the DLBCL tumor microenvironment in patients with different *ALOX15B* expression levels. These patients were classified into two groups based on high and low *ALOX15B* expression (*n* = 8 and *n* = 5, respectively), based on previously described cut-off values. In total, 36,423 (36.3%) B cells, 55,196 (55.0%) T cells, 3,207 (3.2%) NK cells, 3,676 (3.7%) myeloid cells, 1,264 (1.3%) fibroblasts, and 556 (0.5%) endothelial cells were identified and analyzed (Fig. 2A and Supplementary Figure S3A). The proportions of these cell types did not differ significantly between the two groups (Supplementary Figure S3B). B cells were further subdivided into 29 subclusters (Fig. 2B). Due to the heterogeneity of tumor samples, malignant B cells from different patients cluster separately, whereas normal B cells tend to aggregate into similar subclusters [28]. Specifically, the B2, B11, and B20 subclusters, which included cells from multiple patients, were identified as normal B cells (Fig. 2B and Supplementary Figure S3C). These findings were validated by single-cell CNV analysis using B11 as a reference (Supplementary Figure S3D).

We then performed differential gene expression analysis of malignant B cells across the *ALOX15B* subgroups. In the *ALOX15B* high group, we observed the upregulation of the major histocompatibility complex (MHC)-related genes (*HLA-A*, *HLA-B*, *HLA-C*) (Fig. 2C). Enrichment analysis of the DEGs revealed that the apoptosis and class I MHC-mediated antigen processing and presentation pathways were significantly downregulated in malignant B cells from the *ALOX15B* low group (Fig. 2D).

T cells were further classified into eight subclusters. CD4^+^ T cells were categorized into CD4_Tn, CD4_Tcm, CD4_Treg, and CD4_Tfh, whereas CD8^+^ T cells were categorized into CD8_Cyto1, CD8_Cyto2, CD8_Cyto3, and CD8_Cyto4, based on canonical marker genes [29, 30] (Supplementary Figure S4A-B). The two groups showed no statistically significant differences in the proportions of these T-cell subclusters (Supplementary Figure S4C). A similar analysis was performed on myeloid cells, which were classified into six subclusters, namely, M1, M2, cDC1, cDC2, cDC3, and pDC, as identified by canonical marker genes [30, 31] (Supplementary Figure S4D-E). Again, no significant differences in the proportions of these myeloid subclusters were observed between the groups (Supplementary Figure S4F).

We further investigated the interactions between malignant B cells and other cell types within the tumor microenvironment. Analysis of signaling pathway strengths revealed that the prostaglandin pathway was upregulated in the *ALOX15B* low group, whereas the MHC-I pathway was enhanced in the *ALOX15B* high group (Fig. 2E). The transporter associated with antigen processing 1 (TAP1) plays a pivotal role in regulating the assembly and stability of MHC-I molecules [32]. Our single-cell transcriptomic results demonstrated that, *TAP1* expression was significantly reduced in the *ALOX15B* low group, as compared to the *ALOX15B* high group (Supplementary Figure S5A).

As the MHC-I pathway is associated with CD8 ^+^ T cells, we focused further on CD8 ^+^ T cells. Similarly, the expression of MHC-related genes (*HLA-A*, *HLA-B*, *HLA-C*) across various subclusters of CD8 ^+^ T cells was also lower in the *ALOX15B* low group than in the *ALOX15B* high group (Fig. 2F). More importantly, we further discovered that the expression level of *TAP1* in malignant B cells was significantly positively correlated with the important MHC-I genes *HLA-A*, *HLA-B*, and *HLA-C* on CD8 ^+^ T cells (Supplementary Figure S5B). These findings suggested that *ALOX15B* affects MHC-I function by modulating *TAP1* expression. By scoring CD8 ^+^ T cells using a cytotoxicity gene set (including *PRF1*, *IFNG*, *GNLY*, *NKG7*, *GZMB*, *GZMA*, *GZMH*, *KLRK1*, *KLRB1*, *KLRD1*, *CTSW*, and *CST7*), we discovered that the cytotoxicity scores of CD8Cyto2 and CD8Cyto3 were lower in the *ALOX15B* low group (Fig. 2G).

In summary, our analysis revealed that patients in the *ALOX15B* low group exhibited upregulation of the prostaglandin signaling pathway and downregulation of the TAP1/MHC-I axis in malignant B cells. Concurrently, the cytotoxic function of CD8 ^+ ^T cells was diminished in the *ALOX15B* low group. Therefore, *ALOX15B* plays a role in immune evasion mechanisms in DLBCL, suggesting its potential as a therapeutic target.

### *ALOX15B* silencing promotes tumor cell proliferation and resistance to doxorubicin

**Fig. 3:**
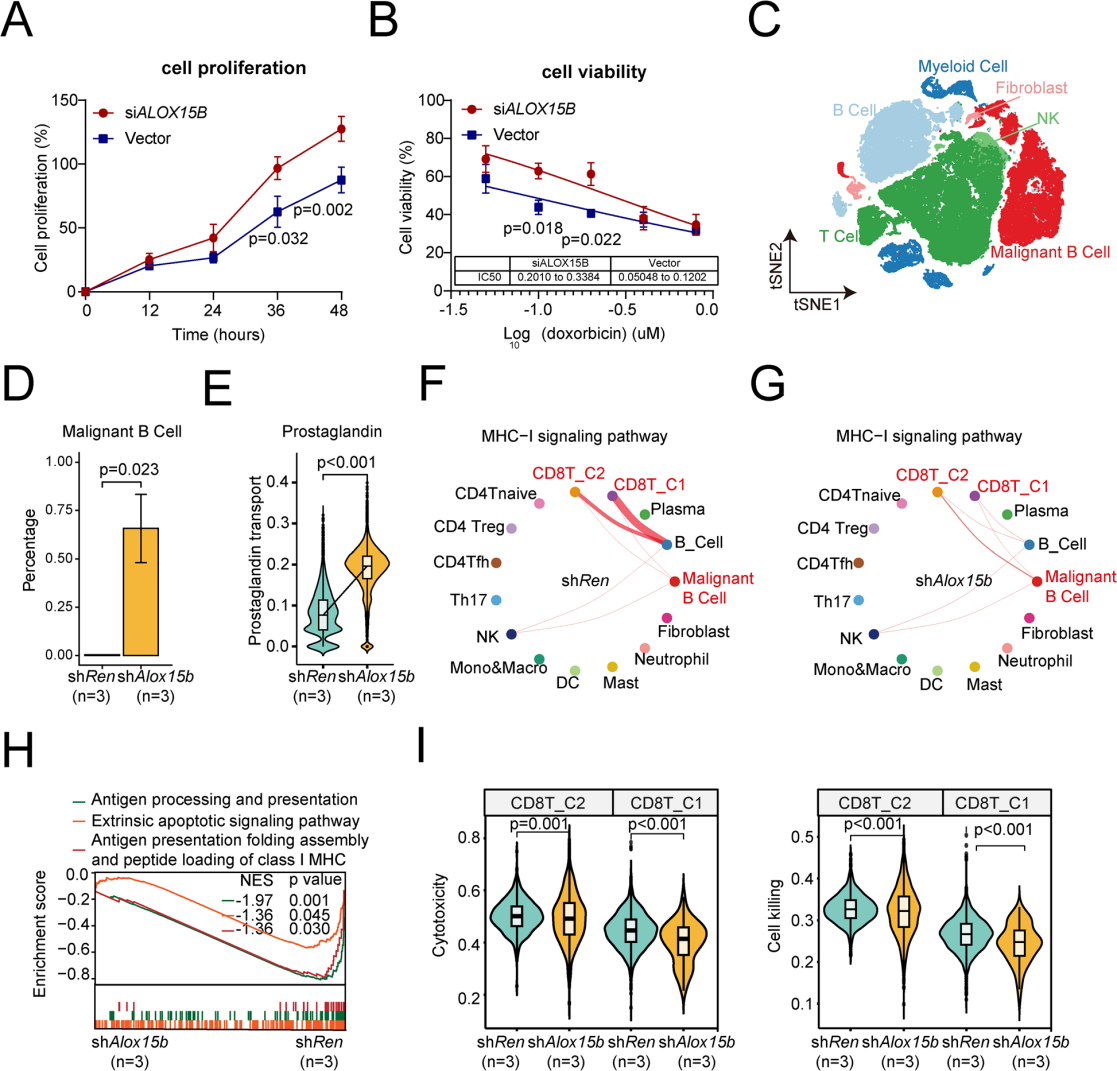
Effects of *ALOX15B* knockdown on cell proliferation, viability, and immune response. **A** Analysis of cell proliferation in si*ALOX15B* and vector control groups. **B** Cell viability assay of the si*ALOX15B* and vector control groups after doxorubicin (Dox) treatment. **C** t-SNE plot of single-cell transcriptomic data showing clustering of cell types, including myeloid cells, fibroblasts, NK cells, T cells, B cells, and malignant B cells. **D** Percentage of malignant B cells in the sh*Alox15b* (*n* = 3) and sh*Ren* (*n* = 3) groups. **E** Expression of prostaglandin transport genes in the sh*Alox15b* and sh*Ren* groups. **F** Network analysis of MHC-I signaling pathways in the sh*Ren* group, with highlighted importance in malignant B cells and CD8 ^+^ T cell clusters. The thickness of the lines represents the probability of cell–cell communication. **G** Network analysis of MHC-I signaling pathways in the sh*Alox15b* group, with highlighted importance in malignant B cells and CD8 ^+^ T cell clusters. **H** GSEA of pathways associated with antigen processing and presentation, extrinsic apoptotic signaling, and MHC-I antigen presentation in the sh*Alox15b* and sh*Ren* models. **I** Cytotoxicity and cell killing assays of CD8 ^+^ T cells in the sh*Alox15b* and sh*Ren* groups

We constructed *ALOX15B* siRNA to further investigate the biological function of *ALOX15B* in DLBCL cell lines. RNA interference with *ALOX15B* expression was performed in two distinct DLBCL subtypes: DB cells (GCB subtype) and U2932 cells (non-GCB subtype). Cell proliferation was assessed at 12, 24, 36, and 48 h post-transfection using CCK-8 assays. The results revealed that at 36 and 48 h, the si*ALOX15B* group showed a significant increase in cell proliferation compared to the vector group (*p* = 0.032 and *p* = 0.002, respectively, Fig. 3A). Given that doxorubicin is a key cytotoxic agent in R-CHOP, we next assessed the sensitivity of the si*ALOX15B* and vector groups to doxorubicin treatment. The cells were exposed to varying concentrations of doxorubicin (0, 0.05, 0.1, 0.2, 0.4, and 0.8 µM) for 48 h. The results indicated that si*ALOX15B* cells exhibited significantly higher viability than the vector group following doxorubicin treatment (Fig. 3B). Meanwhile, we established a murine model of *Alox15b* knockdown (sh*Alox15b*) using a method described in previous reports [18]. We performed scRNA-seq on mice in the control (sh*Ren*) and sh*Alox15b* groups. First, we observed a higher proportion of tumor cells in the sh*Alox15b* group than in the sh*Ren* group (Supplementary Figure S6A-B). Reduced populations of immune cells were associated with sh*Alox15b* tumors, including CD4 ^+^ T and CD8 ^+^ T cells (Fig. 3C-D). Consistent with the findings of the DLBCL cohort shown above, the GSEA results of murine B-cell lymphomas also showed significant activation of prostaglandin transport (Fig. 3E). Furthermore, compared to sh*Ren* controls, MHC-I-dependent interactions between sh*Alox15b* B cells and microenvironmental CD8^ +^ T cells were attenuated (Supplementary Figure S6C-D, Fig. 3F-G). Additionally, compared to the sh*Ren*, the expression of *TAP1* was significantly reduced in the sh*Alox15b* group (Supplementary Figure S6E). Luciferase-reporter assay was then performed, and the results confirmed that si*ALOX15B* could directly repress *TAP1* through transcriptional regulation (Supplementary Figure S6F). Accordingly, antigen presentation (particularly MHC-I signaling) and apoptosis pathways were suppressed in sh*Alox15b* tumor cells compared to that in sh*Ren* control cells (Fig. 3H). Notably, CD8 ^+^ T cells showed marked downregulation of non-classical MHC-I molecules, suggesting a dual defect in both antigen presentation and innate-like T cell recognition pathways in the sh*Alox15b* group (Supplementary Figure S6G). Meanwhile, CD8 ^+^ T cells in the sh*Alox15b* group exhibited significantly reduced cytotoxicity and killing activity compared to the sh*Ren* group (Fig. 3I). Taken together, these results suggested that silencing *Alox15b* in tumors leads to suppressed apoptosis, enhanced prostaglandin E activation, disruption of MHC-I antigen presentation, and thus promotion of an immunosuppressive microenvironment with DLBCL.

### Tucidinostat restores *ALOX15B* expression and enhances cell apoptosis through the TAP1/MHC-I axis in an HDAC1/HDAC2-mediated manner

**Fig. 4:**
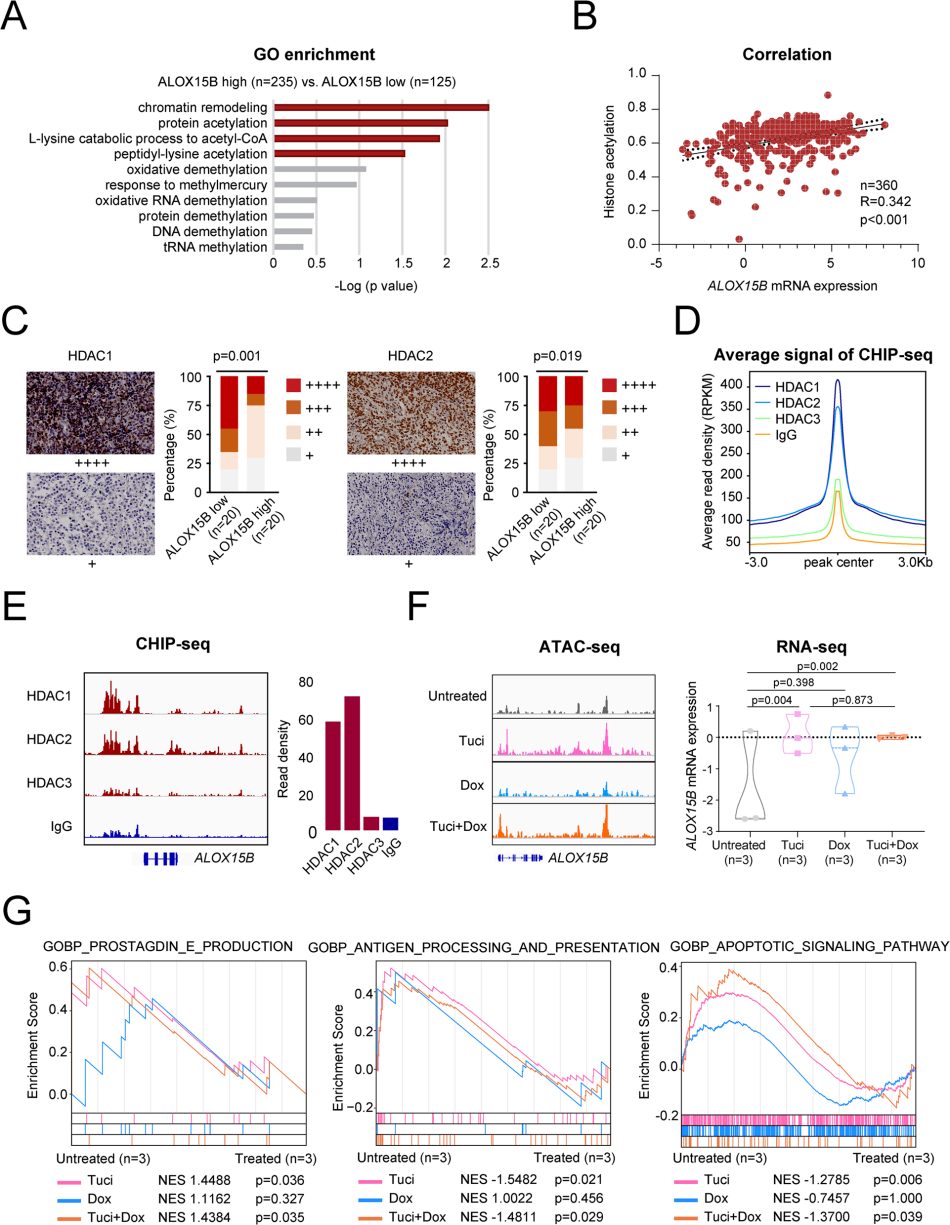
Tucidinostat restores *ALOX15B* expression and enhances lymphoma cell apoptosis through modulating PGE2 production as well as antigen processing and presentation pathways in an HDAC1/HDAC2-dependent manner. **A** Gene ontology (GO) enrichment analysis comparing the high (*n* = 235) and low (*n* = 125) *ALOX15B* expression groups. **B** Correlation between *ALOX15B* mRNA expression and histone acetylation in 360 samples. **C** Immunohistochemical staining of HDAC1 and HDAC2 in the high and low *ALOX15B* expression groups. The percentage of cells with varying levels of expression (from + to + + + +) was quantified, showing a significant difference in HDAC1 and HDAC2 expression between the groups (*p* = 0.001 for HDAC1 and *p* = 0.019 for HDAC2). **D** Average read density from ChIP-seq analysis at the binding locus for HDAC1, HDAC2, and HDAC3, as well as the IgG control. **E** ChIP-seq data showing the binding density of HDAC1, HDAC2, HDAC3, and IgG control at the *ALOX15B* locus. **F** ATAC-seq and RNA-seq data showing changes in chromatin accessibility and *ALOX15B* mRNA expression under tucidinostat (Tuci), doxorubicin (Dox), and the combination of tucidinostat and doxorubicin (Tuci + Dox) treatments. **G** GSEA of pathways associated with prostaglandin E production, antigen processing and presentation, and apoptotic signaling in response to Tuci, Dox, and Tuci + Dox treatment

Based on the above findings, we revealed the prognostic impact of low *ALOX15B* expression in patients with DLBCL and its significant role in tumor cell proliferation and the immune microenvironment modulation. We further explored the regulatory network of *ALOX15B* expression to identify potential targets for optimizing current therapeutic strategies. By comparing the DEGs between patients with high and low *ALOX15B* expression, enrichment analysis revealed significant epigenetic differences between the two groups, particularly in histone acetylation (Fig. 4A). Using the ssGSEA method, we evaluated the acetylation levels in tumors from 360 patients. Pearson’s correlation analysis showed a significant positive correlation between the mRNA expression levels of *ALOX15B* and acetylation levels (R = 0.342, *p* < 0.001) (Fig. 4B). Previous studies have shown that histone acetylation is primarily regulated by class I HDACs, including HDAC1, HDAC2, and HDAC3 [33]. To further investigate the epigenetic regulatory mechanism underlying low *ALOX15B* expression, we assessed the HDAC levels in tumor tissues from patients with high and low *ALOX15B* expression using immunohistochemistry. Figure 4C demonstrated that the proportions of HDAC1 and HDAC2 were significantly higher in the tumors of patients with low *ALOX15B* expression compared to those with high expression. We also conducted ChIP-seq experiments on lymphoma cell lines. The ChIP-seq results from different HDACs (HDAC1, HDAC2, and HDAC3) at the binding locus revealed distinct binding patterns, with HDAC1 and HDAC2 showing the highest read densities compared to HDAC3 and the IgG control (Fig. 4D). HDAC1 and HDAC2 exhibited stronger enrichment at the *ALOX15B* region than HDAC3, consistent with their roles in transcriptional regulation (Fig. 4D). We also consulted a public Gene Expression Omnibus dataset (GSE137666) [34], which confirmed the direct binding of HDAC1 and HDAC2 to the *ALOX15B* region (Supplementary Figure S6H).

Tucidinostat, an oral HDACi, has previously been reported to exhibit significant anti-lymphoma effects. DLBCL cell line DB were treated with tucidinostat, doxorubicin, or their combination for 48 h. Each treatment was performed in triplicate independent experiments. Using ATAC-seq and RNA-seq, we analyzed the effects of these treatments on the chromatin accessibility and transcriptional levels of *ALOX15B*. The results showed that the chromatin accessibility and transcriptional levels of *ALOX15B* were significantly restored after treatment with tucidinostat alone and in combination (Fig. 4F). By comparing the drug-response pathways before and after treatment with tucidinostat, doxorubicin, and the combination, we found that both tucidinostat and the combination treatment inhibited prostaglandin E production, restored antigen presentation, and promoted tumor cell apoptosis (Fig. 4G).

In conclusion, our study revealed that *ALOX15B* expression is regulated by HDAC1/HDAC2-dependent histone acetylation. Tucidinostat restored *ALOX15B* expression, inhibited prostaglandin production, activated antigen presentation, and promotes tumor cell apoptosis.

### Tucidinostat restored *ALOX15B* expression and enhanced in vivo lymphoma cell apoptosis

**Fig. 5:**
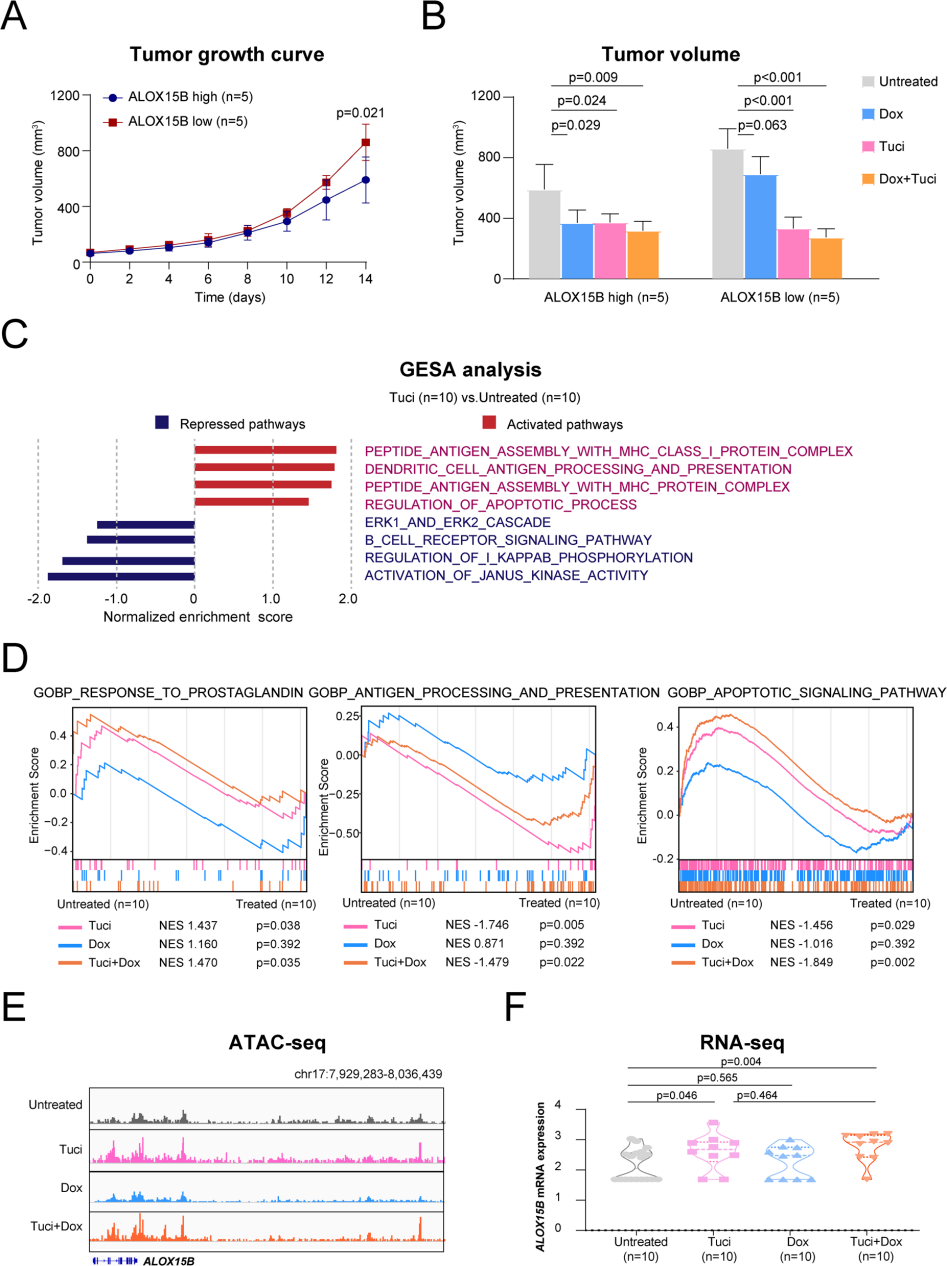
*ALOX15B* expression modulates tumor growth and immune responses in response to various treatments. **A** Tumor growth curve showing a significant increase in tumor volume in the low *ALOX15B* expression group compared to the high *ALOX15B* group over 14 days. **B** Tumor volume measurements in the high and low *ALOX15B* expression groups after treatment with Dox, Tuci, or Tuci + Dox. **C** GSEA comparing the effects of Tuci treatment (*n* = 10) versus untreated conditions (*n* = 10). **D** GSEA of pathways associated with prostaglandin E production, antigen processing and presentation, and apoptotic signaling pathways in the untreated and treated groups (Tuci, Dox, Tuci + Dox). **E** ATAC-seq data showing chromatin accessibility at the *ALOX15B* locus in response to various treatments (untreated, Tuci, Dox, Tuci + Dox). **F** RNA-seq data showing *ALOX15B* mRNA expression in response to Tuci, Dox, and Tuci + Dox treatment

We previously established 10 patient-derived xenograft (PDX) models. The tumor growth curves revealed that tumors in the *ALOX15B* low-expression PDX model proliferated significantly more rapid than those in the *ALOX15B* high-expression group (Fig. 5A). To assess the therapeutic effects of different drug treatments, PDX models were treated with tucidinostat, doxorubicin, and a combination of tucidinostat and doxorubicin. Tumor inhibition analysis demonstrated that the *ALOX15B* low-expression group exhibited reduced sensitivity to doxorubicin, which further validated the results of our in vitro doxorubicin drug sensitivity assays (Fig. 3B). Importantly, *ALOX15B* low-expression PDX mice exhibited a favorable response to tucidinostat, and combination treatment significantly inhibited tumor growth (Fig. 5B). We further investigated the therapeutic response pathways of the tucidinostat, doxorubicin, and combination treatment groups in tumors before and after treatment (Fig. 5C-D). Consistent with our in vitro experiments, both tucidinostat and combination therapy significantly enhanced tumor antigen presentation and promoted tumor cell apoptosis. We also analyzed the effects of these treatments on the chromatin accessibility and transcriptional levels of *ALOX15B* using ATAC-seq and RNA-seq. The results demonstrated that treatment with tucidinostat and combination therapy significantly restored the chromatin accessibility and transcriptional levels of *ALOX15B* expression (Fig. 5E-F).

### Tucidinostat modulates the tumor immune microenvironment by enhancing activation of the Tap1/MHC-I axis in vivo

**Fig. 6:**
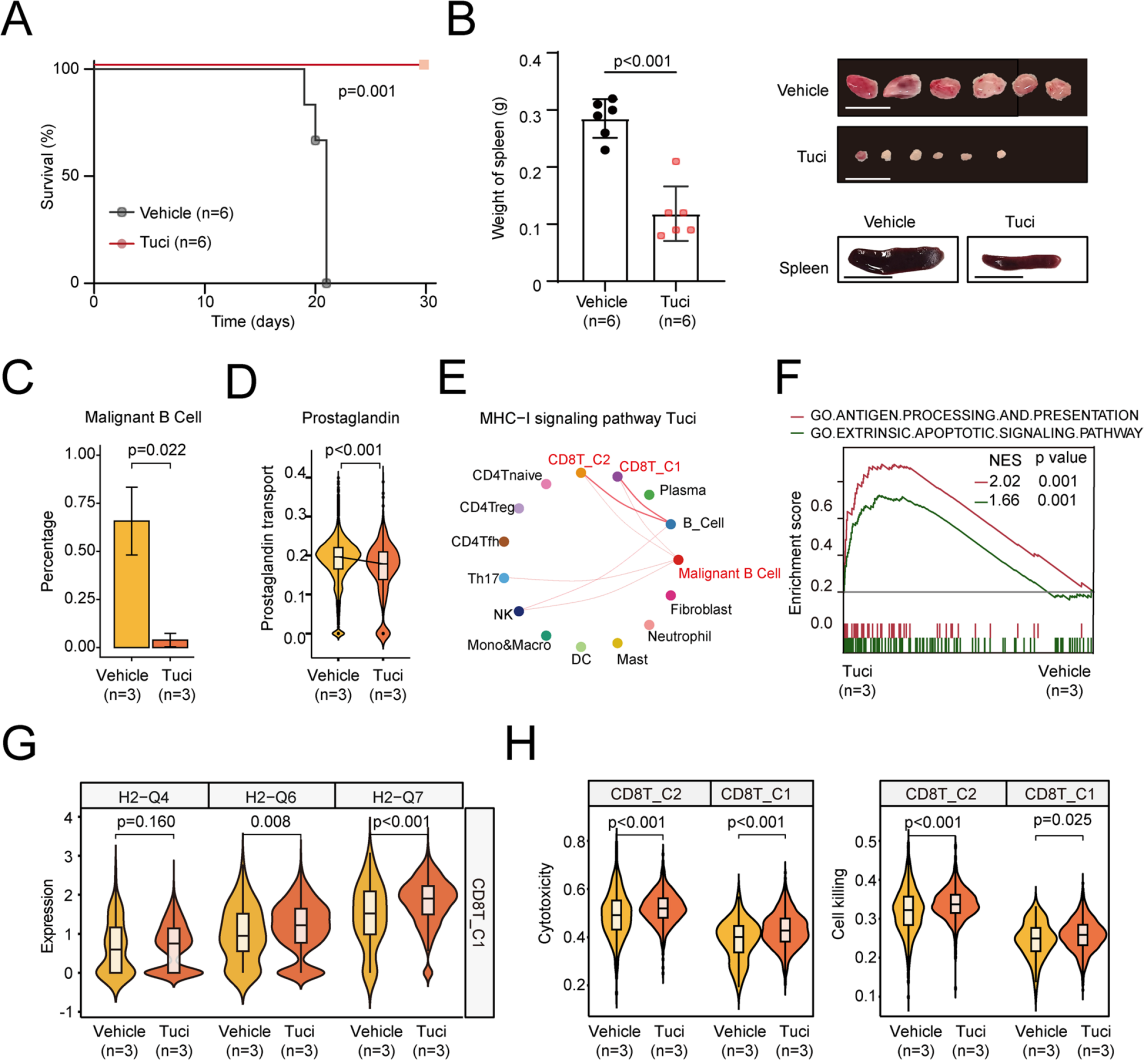
Tucidinostat treatment reduces the tumor burden and modulates the immune response. **A** Kaplan–Meier tumor-free survival curves between the vehicle- and Tuci-treated groups, *p* = 0.001 (log-rank test). **B** Spleen weight measurement and representative images of spleen size and subcutaneous tumor tissues from the xenograft models in the vehicle- and Tuci-treated groups. *p* < 0.001 (log-rank test); scale bar: 5 mm. **C** Percentage of malignant B cells in the vehicle- and Tuci-treated groups. **D** Expression of prostaglandin transport genes in the sh*Alox15b* and Tuci-treated groups. **E** Network analysis of MHC-I signaling pathways in the Tuci-treated groups, with highlighted importance in malignant B cells and CD8 ^+^ T cell clusters. The thickness of the lines represents the probability of cell–cell communication. **F** GSEA comparing the effects of Tuci treatment (*n* = 3) versus untreated conditions (*n* = 3). **G** Violin plots showing MHC-I expression in CD8 ^+^ T cells for following subtypes: H2-Q4, H2-Q6, and H2-Q7 in vehicle- and Tuci-treated groups. **H** Cytotoxicity and cell killing assays of CD8 T_C1 and CD8T_C2 cells in the vehicle and Tuci-treated groups

To evaluate the impact of tucidinostat on the tumor microenvironment of *ALOX15B*-deficient lymphoma, we established an *Alox15b*-knockdown lymphoma model in immunocompetent C57BL/6 mice, as reported previously [35]. Briefly, B progenitor cells isolated from the bone marrow of C57BL/6 mice were co-transduced with sh*Alox15b* and *Myc* cDNA, followed by transplantation into irradiated wild-type C57BL/6 mice. One week after transplantation, the recipient mice were randomly divided into two groups: one group was treated with vehicle, and the other group was treated with tucidinostat (12.5 mg/kg/day). Compared to vehicle-treated mice that developed lymphomas within 3 weeks, tucidinostat-treated mice were healthy during the observed period (Fig. 6A-B).

To determine whether tucidinostat reverses *Alox15b* knockdown-mediated reprogramming of the tumor microenvironment, we performed 10 × Genomics scRNA-seq of lymph nodes harvested from recipient mice treated with vehicle or tucidinostat for 1 week. The results revealed that tucidinostat treatment significantly reduced the proportions of tumor cells compared to the vehicle group (Supplementary Fig. 6I), concurrently suppressing prostaglandin signaling while enhancing antigen presentation and apoptotic pathway (Fig. 6C-F). MHC-I signaling networks were restored in tucidinostat-treated cells but not in the vehicle group (Fig. 6E). *Tap1* expression and non-classical MHC-I molecules were significantly upregulated in tucidinostat-treated CD8 ^+ ^T cells (Supplementary Figure S6J, Fig. 6G), which exhibited significantly enhanced cytotoxicity and cytotoxic activity compared to the vehicle group (Fig. 6H). In conclusion, our results in an immunocompetent mouse model supported that tucidinostat inhibited the abnormally activated prostaglandin E pathway and restored MHC-I-related antigen presentation pathways. This, in turn, improved the immune function of CD8 ^+ ^T cells within the tumor immune microenvironment, enhanced anti-tumor cytotoxicity, and promoted tumor cell apoptosis. The *ALOX15B*/*TAP1*/MHC-I axis was markedly increased both in vitro and in vivo after HDACi treatment, as revealed by qRT-PCR, immunoblotting, and immunohistochemistry (Supplementary Figure S7). These findings are consistent with clinical outcomes observed in a phase II trial (NCT02753647) of tucidinostat plus R-CHOP (CR-CHOP) in newly diagnosed DLBCL patients (Supplementary Figure S8). Survival analysis revealed that the addition of tucidinostat improved the outcome of patients with low *ALOX15B* expression who experienced poor response to RCHOP, as *ALOX15B* expression no longer influenced the PFS of patients receiving CR-CHOP.

## Discussion

In this study, we identified *ALOX15B*, a gene located on the 17p region, as a critical factor in predicting the prognosis of patients with DLBCL. The 17p^−^ is associated with poor prognosis in various lymphoid malignancies, including multiple myeloma [4], chronic lymphocytic leukemia [5], mantle cell lymphoma [6], and DLBCL [36]. In addition to *TP53*, the well-known tumor suppressor gene located on 17p [3], we demonstrated that low expression of *ALOX15B* is related to activation of the prostaglandin pathway, attenuation of TAP1/MHC-I axis, which in turn promotes lymphoma progression.

ALOX15B is an enzyme involved in the metabolism of arachidonic acid, a polyunsaturated fatty acid that can be metabolized through multiple pathways, including the cyclooxygenase pathway responsible for prostaglandin synthesis [7]. Low ALOX15B expression leads to the accumulation of arachidonic acid and upregulation of the cyclooxygenase pathway, particularly the production of PGE2. PGE2 is a bioactive lipid that plays an essential role in the tumor microenvironment by promoting tumor growth, angiogenesis, and immune evasion [37]. In melanoma, PGE2 can enhance tumor progression by modulating the immune response through its receptors (EP1-EP4), thereby inducing the dysfunction of cDC1 and inactivating CD8 ^+^ T cells, leading to immunosuppression and a more favorable environment for tumor growth [38].

Concerning the tumor microenvironment, scRNA-seq results revealed that low *ALOX15B* expression was associated with decreased MHC-I molecule in malignant B cells. This reduction in MHC-I expression significantly impairs the communication between tumor B cells and T cells, leading to an immunosuppressive microenvironment. This finding provided a mechanistic explanation for the poor prognosis observed in patients with low *ALOX15B* expression who undergo R-CHOP immunochemotherapy. Furthermore, TAP1, which is responsible for efficiently presenting antigens to MHC-I molecules and the assembly of MHC-I molecules [39, 40], was expressed at low levels in both patients and murine models with low *ALOX15B* expression. The luciferase reporter assay demonstrated that ALOX15B could interact with the promoter of *TAP1*, providing insights into the mechanism of ALOX15B-regulating *TAP1* transcription and subsequently MHC-I expression. Consistent with our observations, TAP1-deficient mice exhibit a significant reduction in MHC-I expression and the number of CD8 ^+^ T cells, which impairs host immune responses [41]. Specifically, the downregulation of MHC-I molecules on tumor B cells limits the ability of T cells to recognize and kill tumor cells, thereby facilitating tumor immune evasion [42]. This immunosuppressive landscape within the tumor microenvironment is a critical factor contributing to the poor response to immunochemotherapy in these patients. Future therapeutic strategies targeting the restoration of MHC-I expression or the enhancement of T cell activity may improve outcomes in patients with low *ALOX15B* expression.

However, further investigation showed that the low expression of *ALOX15B* in lymphoma cells was independent of 17p^−^, and ChIP-seq analysis revealed that the expression of *ALOX15B* was modulated by HDACs, specifically HDAC1 and HDAC2. HDACs modulate gene expression by deacetylating histones, thereby influencing chromatin structure and accessibility [43]. We further demonstrated that restoring *ALOX15B* expression using the HDACi tucidinostat retarded lymphoma cell growth both in vitro and in vivo by inhibiting the PGE2 pathway, enhancing antigen presentation, and promoting lymphoma cell apoptosis. Tucidinostat is a novel benzamide HDAC inhibitor that inhibits class I HDAC1, HDAC2, HDAC3, and class IIb HDAC10 [44]. In this study, we demonstrated that in DLBCL, tucidinostat increased histone acetylation, inducing an open chromatin state to improve the binding of transcription factors to the *ALOX15B* promoter, leading to elevated *ALOX15B* expression [45]. These findings provided insights into the ability of epigenetic agents to restore the expression of genes located on 17p in 17p^−^tumors.

## Conclusions

In conclusion, our study provided evidence for the critical role of *ALOX15B* in DLBCL pathogenesis. The downregulation of *ALOX15B* is closely related to immune suppression, poor prognosis, and chemoresistance, highlighting its potential as a valuable biomarker for identifying patients at high risk of treatment failure. Additionally, our findings suggested that tucidinostat could restore *ALOX15B* expression in an HDAC1/HDAC2-dependent manner, suggesting a promising therapeutic strategy for improving treatment outcomes in patients with DLBCL.

## Supplementary Information

Supplementary Material 1: Supplementary Figure S1. Flowchart of patient selection and analysis. This flowchart outlines the process of patient selection and analysis in the study. A total of 360 patients with bulk-RNA sequencing data were included. These patients were categorized into two groups based on their treatment regimen: R-CHOP cohort (n=329) and CR-CHOP cohort (n=31). Supplementary Figure S2. Analysis of genes located on the 17p.13 region. (A) Scatter plot showing the relationship between gene expression levels and PFS for 195 genes located on the 17p.13 region. The genes* ALOX15B*, *TAX1BP3*, *MYH8*, *KIF1C*, and *PITPNM3* are highlighted in red, indicating that their low expression levels are significantly associated with PFS, based on the log_2_ (fold change) and −log_10_ (*p*-value). (B) Table presenting the area under the curve (AUC) values for the genes *ALOX15B*,* TAX1BP3*,* MYH8*,* PITPNM3*, and *KIF1C*, along with standard error and asymptotic significance. (C) Box plot comparing the mRNA expression levels of *ALOX15B* in patients with and without the 17p deletion (17p^-^). The expression levels of *ALOX15B* are shown for 17p^-^ (n=21) and non-17p^-^ (n=332) groups, with no significant difference (p=0.583). Supplementary Figure S3. Identification of distinct cell types within the tumor microenvironment of patients with diffuse large B-cell lymphoma. (A) Density plot depicting the expression of marker genes across different cell types. (B) Proportions of each cell type in patients grouped by different *ALOX15B* expression levels. (C) t-SNE plot showing the distribution of B cells across various samples. (D) Heatmap representing copy number variations (CNVs) for each B cell subcluster (reference: cluster B11; red indicates amplifications, blue indicates deletions). (E) Proportion of malignant B cells within the total B cell population across patients with different *ALOX15B* groups. Supplementary Figure S4. Analysis of T cells and myeloid cells in the tumor microenvironment. (A) t-SNE plot illustrating the distribution of T cells across different subclusters. (B) Dot plot showing the expression of marker genes for T cell subclusters. (C) Proportions of CD4^+^ and CD8^+^ T cell subclusters in patients classified by different *ALOX15B* expression levels. (D) t-SNE plot depicting the distribution of myeloid cells across various subclusters. (E) Density plot of marker gene expression in different myeloid subclusters. (F) Proportions of myeloid subclusters in patients with different *ALOX15B* groups. Supplementary Figure S5. Analysis of *TAP1* expression and its correlation with MHC-I expression in CD8^+^ T cells. (A) Box plot showing the mRNA expression of *TAP1* in patients with low and high *ALOX15B* expression. (B) Correlation analysis between* TAP1* expression in malignant B cells and MHC-I expression in CD8^+^ T cells for three HLA subtypes: (left) HLA-A, (middle) HLA-B, and (right) HLA-C. Supplementary Figure S6. Immune cell identification and analysis of TAP1 regulation. (A) Dot plot showing the normalized expression of various genes across different cell types. The size of the dots indicates the percentage of cells expressing each gene, while the color intensity represents the normalized expression level of each gene. (B) Stacked bar plot showing the relative abundance of different cell types in sh*Ren* and sh*Alox15b* conditions. (C) Dot plot showing the expression of various genes across subtypes of CD8 T cells, CD4 T cells, Th17 cells, and other immune cells. The size of the dots indicates the percentage of cells expressing each gene, and the color represents the average expression level. (D) UMAP (Uniform Manifold Approximation and Projection) plot showing the clustering of different T cell subtypes, including CD4 and CD8 subsets, Th17, and CD4 Treg cells. (E) Violin plot showing the mRNA expression of *Tap1* in *Ren* and sh*Alox15b* murine models. (F) Bar chart depicting the relative luciferase activity of the *TAP1* promoter in DB and U2932 cell lines with either a vector or si*ALOX15B*. (G) Violin plots showing MHC-I expression in CD8^+^ T cells for following subtypes: H2-Q4, H2-Q6, and H2-Q7 in *Ren* and sh*Alox15b* groups. (H) ChIP-seq analysis from a public Gene Expression Omnibus data (GSE137666) showing Hdac1 and Hdac2 binding at the *Alox15b* locus, with input control shown at the bottom. (I) Stacked bar plot showing the relative abundance of different cell types in vehicle and Tuci-treated groups. (J) Violin plot showing the mRNA expression of *TAP1* in sh*Alox15b* murine models under Tuci trearment. Supplementary Figure S7. Tucidinostat restores ALOX15B expression and enhances the Tap1/MHC-I axis both in vitro and in vivo. (A) qRT-PCR analysis of *TAP*1, *HLA-A*, *HLA-B*, and *HLA-C* mRNA expression in lymphoma cell lines DB and U2932 treated with Tuci or untreated control. (B) Immunoblot analysis showing increased TAP1 and HLA-ABC protein levels following Tuci treatment in si*ALOX15B* lymphoma cell lines DB and U2932. (C-D) Immunoblot analysis of TAP1 and HLA-ABC expression in subcutaneous tumor tissues from xenograft models in the indicated groups (sh*Ren*, sh*Alox15b*, and sh*Alox15b *+ Tuci), with three replicates per group. (E) qRT-PCR and immunoblot showing increased *ALOX15B* expression in lymphoma cell lines DB and U2932 following Tuci treatment. (F) qRT-PCR and immunohistochemical staining demonstrating upregulation of *ALOX15B* in tumor tissues from PDX models treated with Tuci compared with untreated controls (*n* = 10 per group). Scale bar, 50 μm. Data are presented as mean ± SEM. Supplementary Figure S8. Survival analysis of *ALOX15B* expression in CR-CHOP cohort. (A-B) PFS (A) and OS (B) of patients in the CR-CHOP cohort categorized by high (*n* = 19) and low (*n* = 12) *ALOX15B* expression.

Supplementary Material 2.

## Data Availability

Raw scRNA-seq generated in this study have been deposited at the GSA-Human database (Accession number HRA007235, https://ngdc.cncb.ac.cn/gsa-human/s/746Ca9Hq). The scRNA-seq data of mouse in this study are deposited in NCBI GEO under accession number GSE294509. Published bulky RNA sequencing data from previous studies were available on the National Omics Data Encyclopedia (https://www.biosino.org/node) under the project OEP001143 and OEP001242. Data are open to the scientific and medical community. Proposals requesting individual participant data that underlie the results reported in this article (after de-identification) can be sent to Prof. Wei-Li Zhao, zhao.weili@yahoo.com. A steering committee involving all principal investigators will evaluate the request and make the decision before sending the database to any academic partners.

## Abbreviations

AA: Arachidonic acid
ALOX15B: Arachidonate 15-lipoxygenase type B
HDAC: Histone deacetylase
DLBCL: Diffuse large B-cell lymphoma
R-CHOP: Rituximab, cyclophosphamide, doxorubicin, vincristine, and prednisone
17p^-^: Chromosome 17p deletion
PFS: Progression-free survival
GSEA: Gene set enrichment analysis
SsGSEA: Single -sample GSEA
GCB: Germinal-center B-cell-like
DE: BCL2/MYC double expression
FBS: Fetal bovine serum
siRNAs: Small interfering RNAs
scRNA-seq: Single-cell RNA sequencing
PBS: Phosphate-buffered saline
CNV: Copy number variation
DEGs: Differentially expressed genes
ChIP-seq: Chromatin immunoprecipitation sequencing
ROC: Receiver operating characteristic
OS: Overall survival
GO: Gene ontology
MHC: Major histocompatibility complex
PDX: Patient-derived xenograft
