# Supplementary material for: Low expression of ALOX15B modulates immunosuppressive tumor microenvironment in diffuse large B-cell lymphoma via the TAP1/MHC-I axis
Source: J Exp Clin Cancer Res. 2026 Jan 12;45:43. doi: 10.1186/s13046-025-03613-2 (PMC12888490; doi:10.1186/s13046-025-03613-2)

Supplementary Figure S1

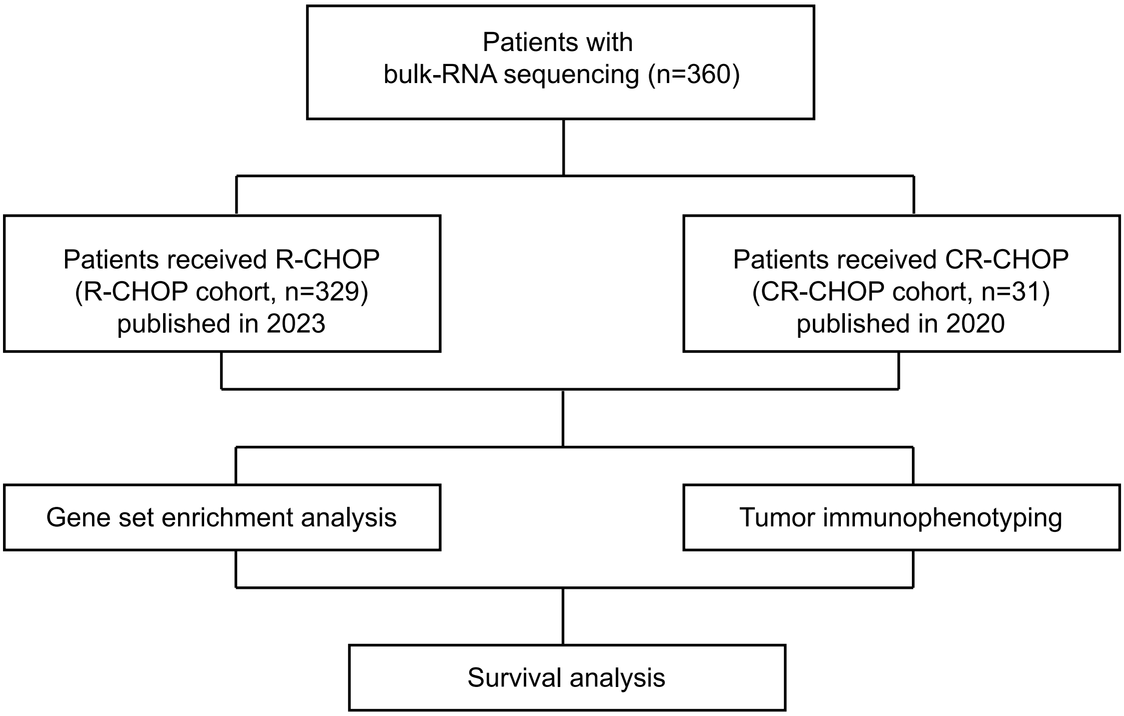

Supplementary Figure S2

A

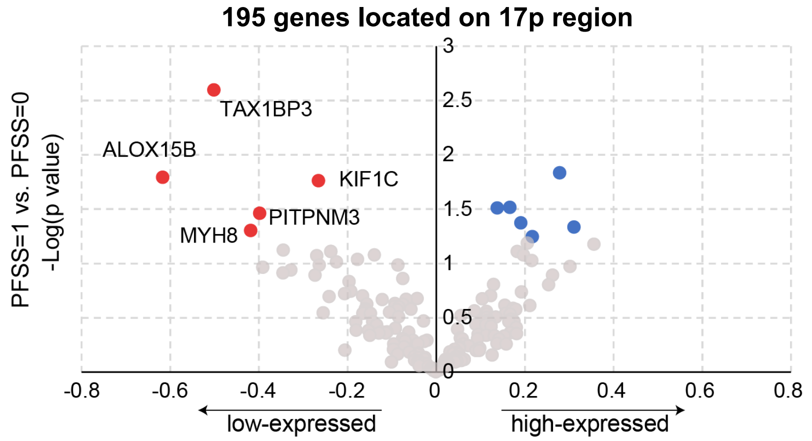

B

Area Under the Curve

Test Result Variable(s)	Area	Std. Error[a]	Asymptotic Sig.[b]	Asymptotic 95% Confidence Interval	
				Lower Bound	Upper Bound
ALOX15B	.413	.032	.009	.350	.477
TAX1BP3	.392	.032	.001	.331	.454
MYH8	.450	.033	.136	.386	.514
PITPNM3	.441	.033	.078	.377	.505
KIF1C	.440	.032	.075	.377	.504

The test result variable(s): ALOX15B, TAX1BP3, MYH8, PITPNM3, KIF1C has at least one tie between the positive actual state group and the negative actual state group. Statistics may be biased.

a. Under the nonparametric assumption

b. Null hypothesis: true area = 0.5

C

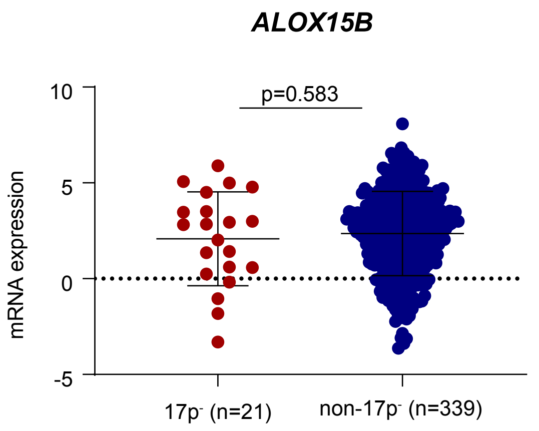

Supplementary Figure S3

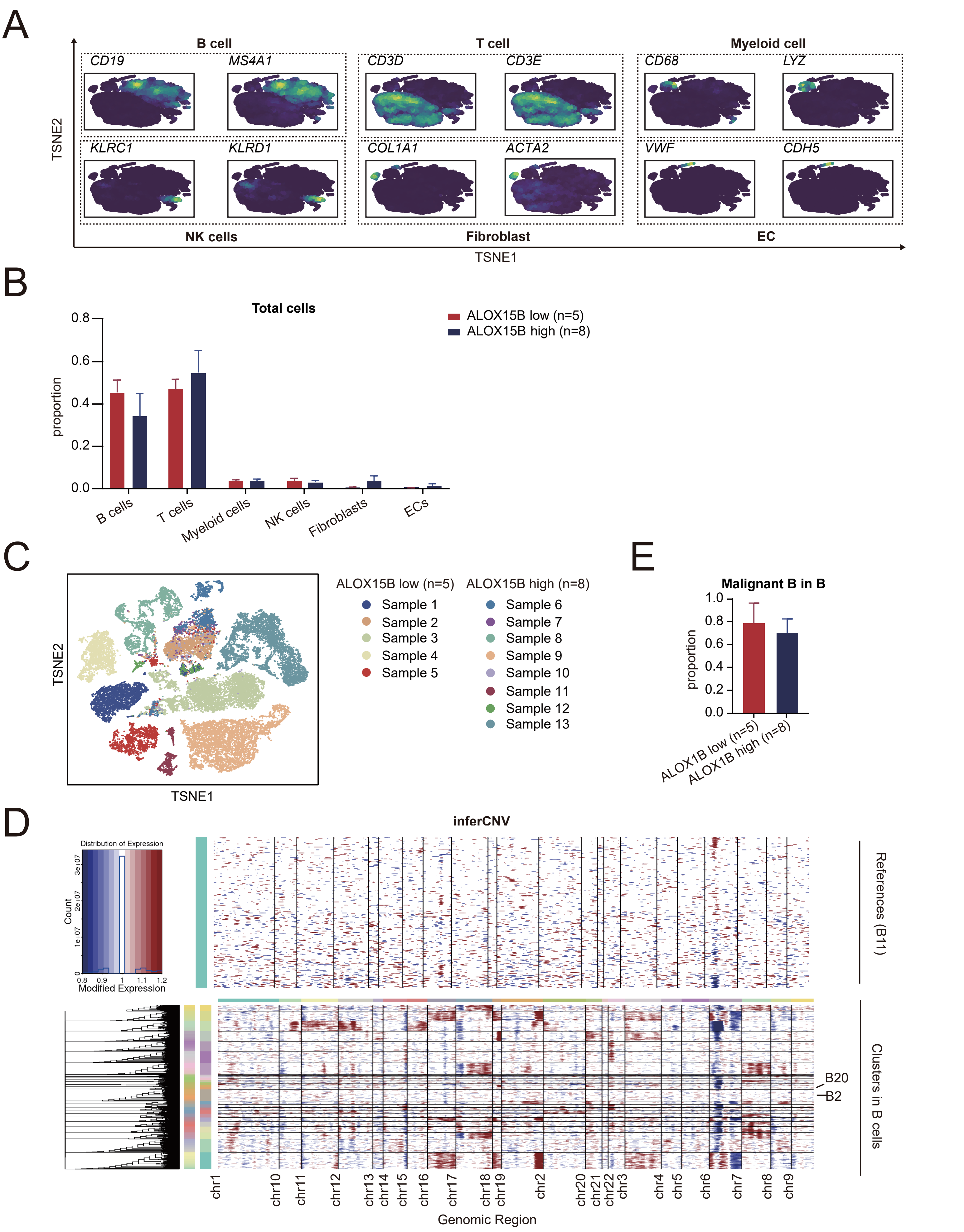

Supplementary Figure S4

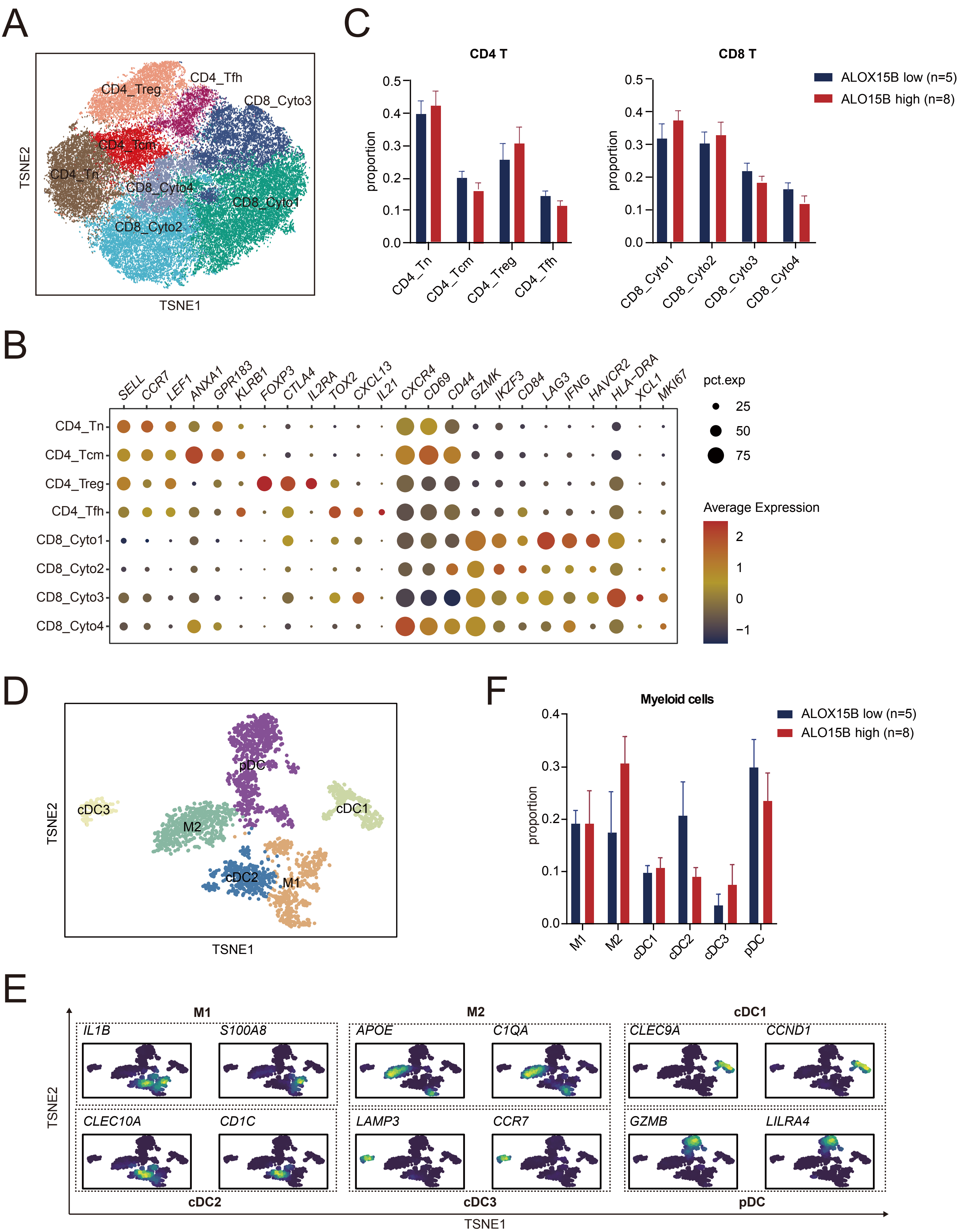

Supplementary Figure S5

A

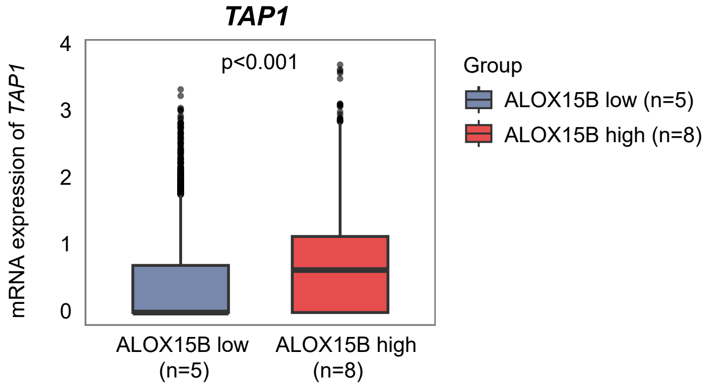

B

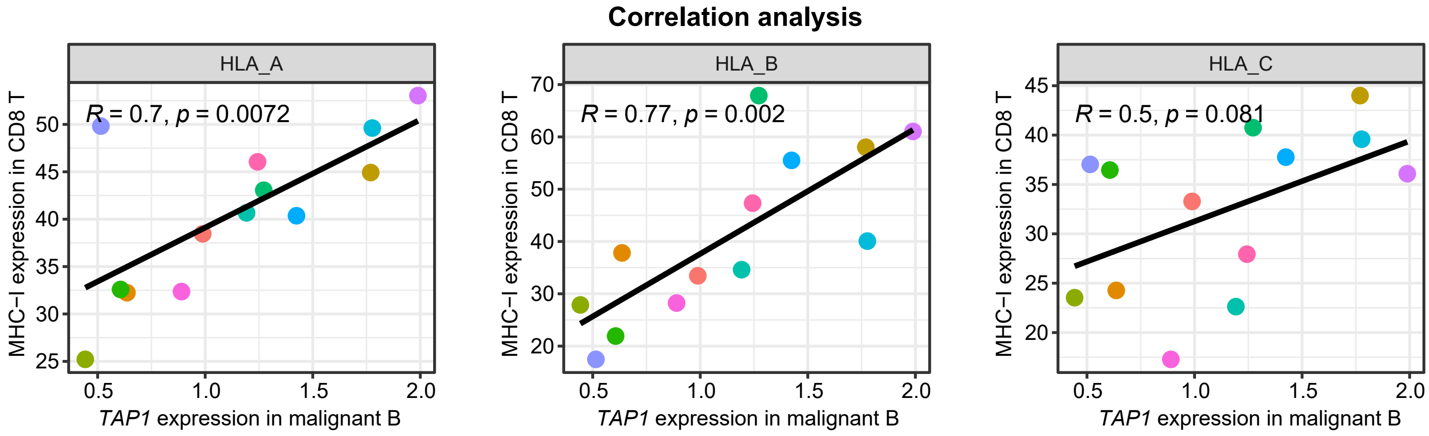

Supplementary Figure S6

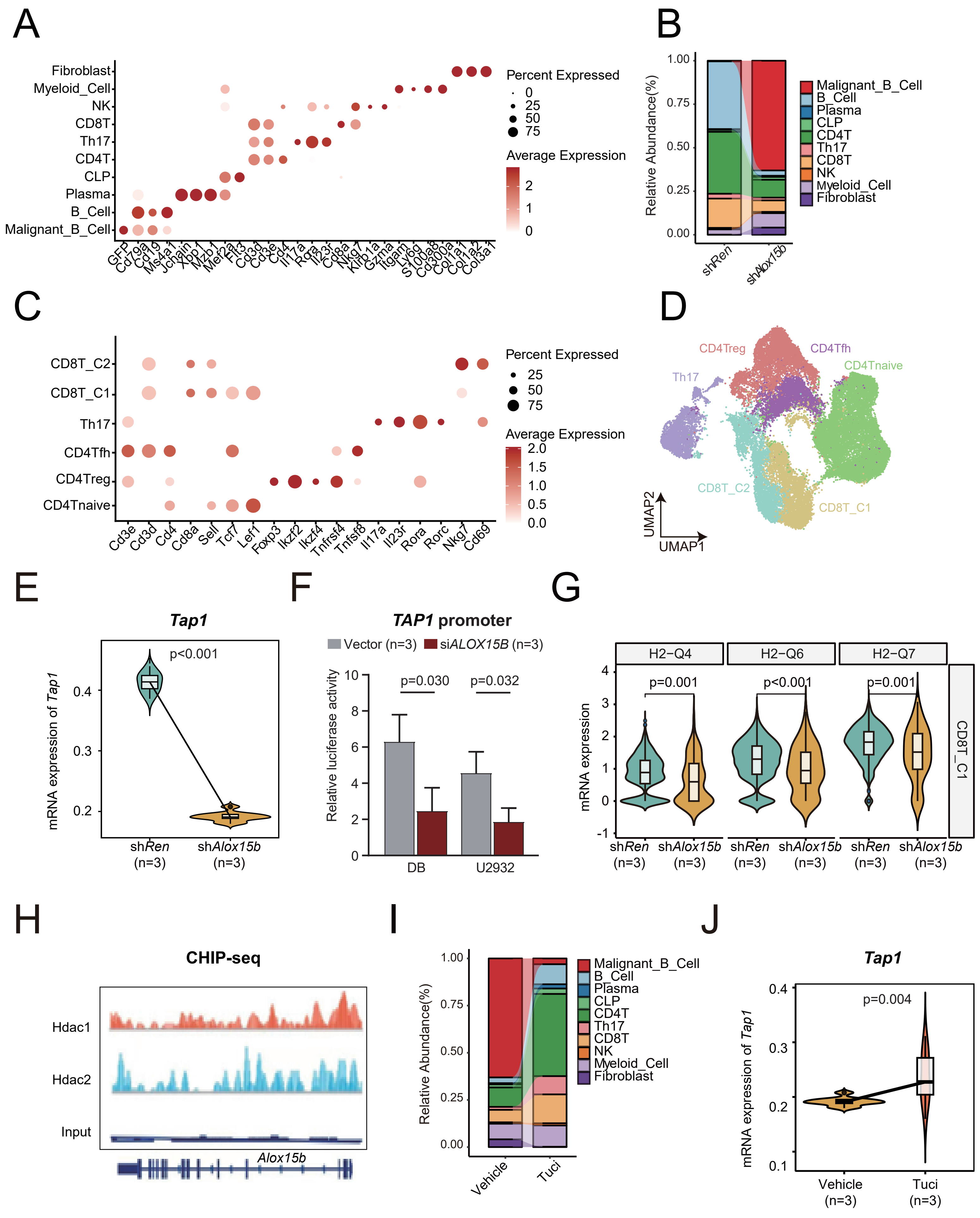

Supplementary Figure S7

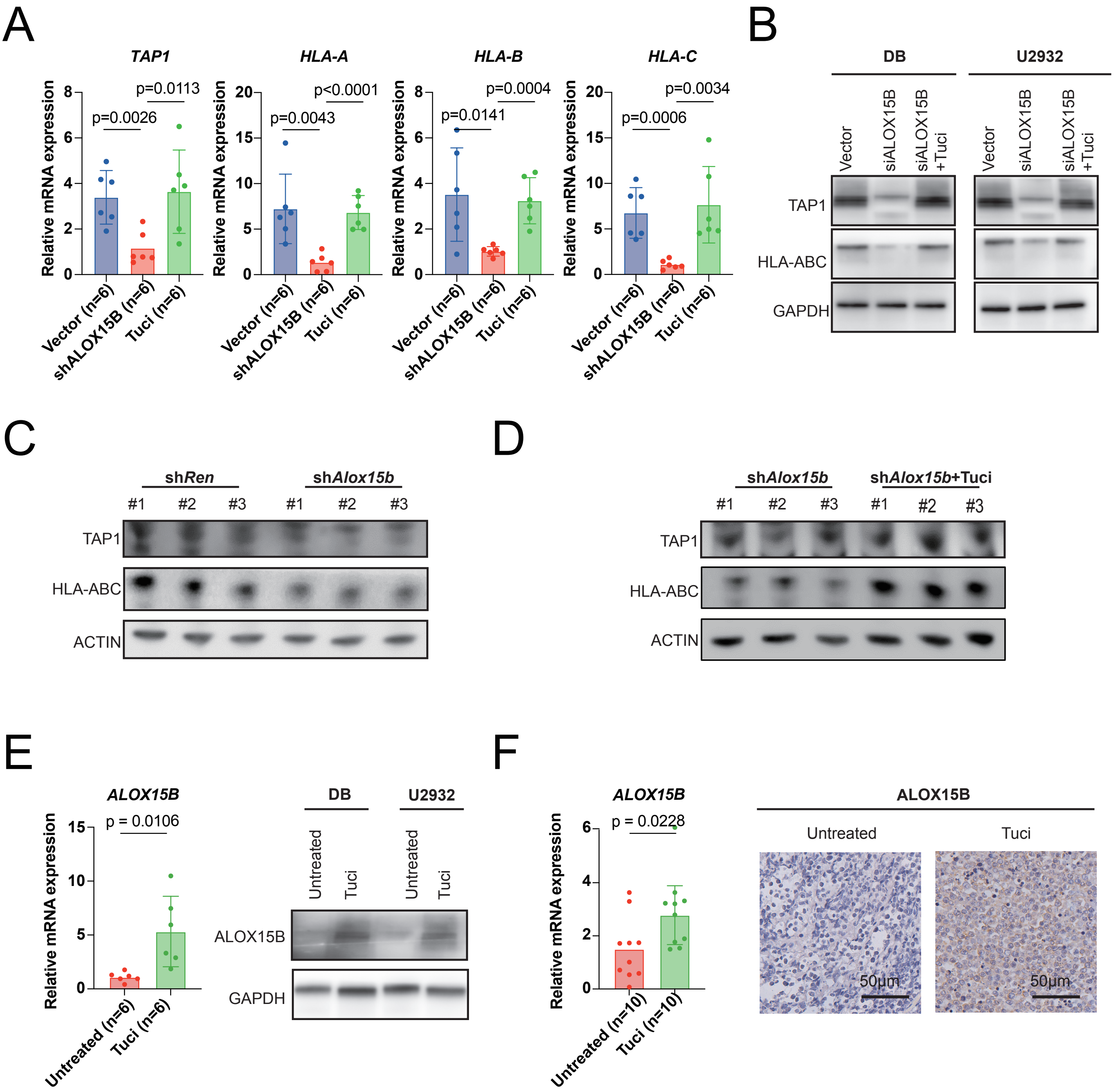

Supplementary Figure S8

A

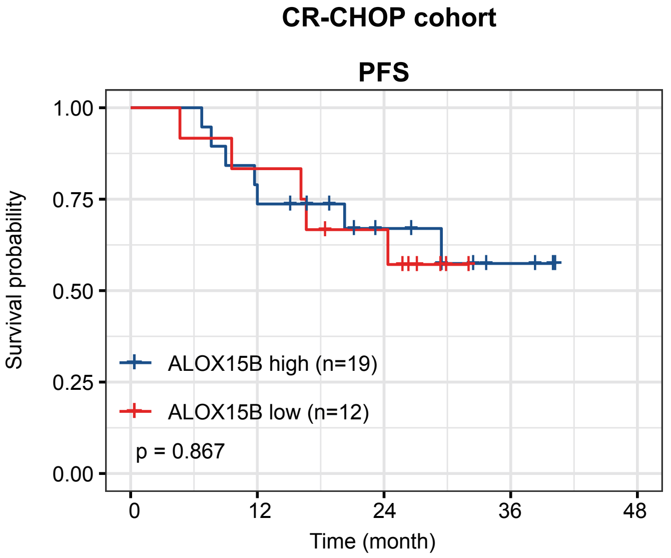

B

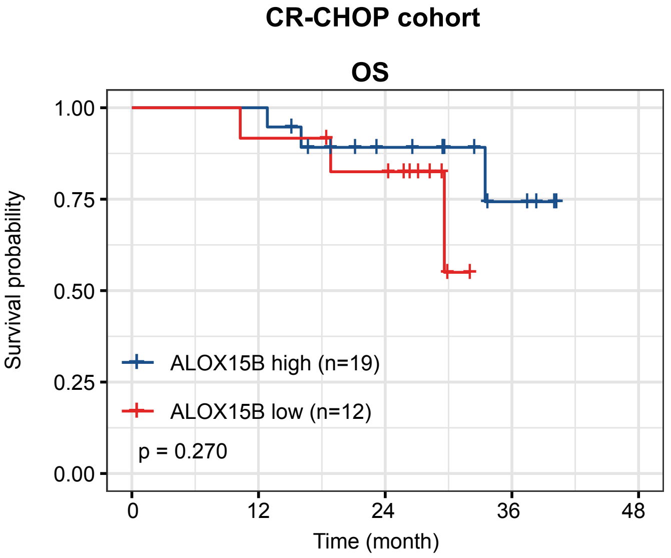

Supplement: Supplementary file 1 — Supplementary Material 1: Supplementary Figure S1. Flowchart of patient selection and analysis. This flowchart outlines the process of patient selection and analysis in the study. A total of 360 patients with bulk-RNA sequencing data were included. These patients were categorized into two groups based on their treatment regimen: R-CHOP cohort (n=329) and CR-CHOP cohort (n=31). Supplementary Figure S2. Analysis of genes located on the 17p.13 region. (A) Scatter plot showing the relationship between gene expression levels and PFS for 195 genes located on the 17p.13 region. The genes ALOX15B, TAX1BP3, MYH8, KIF1C, and PITPNM3 are highlighted in red, indicating that their low expression levels are significantly associated with PFS, based on the log2 (fold change) and −log10 (p-value). (B) Table presenting the area under the curve (AUC) values for the genes ALOX15B, TAX1BP3, MYH8, PITPNM3, and KIF1C, along with standard error and asymptotic significance. (C) Box plot comparing the mRNA expression levels of ALOX15B in patients with and without the 17p deletion (17p-). The expression levels of ALOX15B are shown for 17p- (n=21) and non-17p- (n=332) groups, with no significant difference (p=0.583). Supplementary Figure S3. Identification of distinct cell types within the tumor microenvironment of patients with diffuse large B-cell lymphoma. (A) Density plot depicting the expression of marker genes across different cell types. (B) Proportions of each cell type in patients grouped by different ALOX15B expression levels. (C) t-SNE plot showing the distribution of B cells across various samples. (D) Heatmap representing copy number variations (CNVs) for each B cell subcluster (reference: cluster B11; red indicates amplifications, blue indicates deletions). (E) Proportion of malignant B cells within the total B cell population across patients with different ALOX15B groups. Supplementary Figure S4. Analysis of T cells and myeloid cells in the tumor microenvironment. (A) t-SNE plot [file 13046_2025_3613_MOESM1_ESM.pdf]
