# Supplementary material for: Low expression of ALOX15B modulates immunosuppressive tumor microenvironment in diffuse large B-cell lymphoma via the TAP1/MHC-I axis
Source: J Exp Clin Cancer Res. 2026 Jan 12;45:43. doi: 10.1186/s13046-025-03613-2 (PMC12888490; doi:10.1186/s13046-025-03613-2)
Supplement: Supplementary file 2 — Supplementary Material 2. [file 13046_2025_3613_MOESM2_ESM.docx]

**Supplementary Table S1.**

Sequences of primers for RT-PCR.

| Name | Forward | Reverse |
| --- | --- | --- |
| *ALOX15B* | GACCCTGCTATACCAGAGCC | ACCAGTCCCACTTGTCATCAG |
| *TAP1* | CTGGGGAAGTCACCCTACC | CAGAGGCTCCCGAGTTTGTG |
| *HLA-A* | AAAAGGAGGGAGTTACACTCAGG | GCTGTGAGGGACACATCAGAG |
| *HLA-B* | GGGATGGCGAGGACCAAAC | ACAGCTCCGATGACCACAAC |
| *HLA-C* | CCATGAGGTATTTGTGGACCG | TCTCGGACTCTCGTCGTCG |
| *GAPDH* | GGAGCGAGATCCCTCCAAAAT | GGCTGTTGTCATACTTCTCATGG |
| *Tap1* | TTCCCTCAGGGCTATGACAC | CTGTCGCTGACCTCCTGAC |
| *H2-Q4* | GGATGGAACCTTCCAGAAGTGG | GCCATGTTGGAGACAGTGGATG |
| *H2-Q6* | GAGATACTACAACCAGAGCAAGG | TTTCAGGTCTTCGTTCAGGG |
| *H2-Q7* | GAGCAGGCTGGTATTGCAGAG | CACCATAAGACCTGGGGTGA |
| *Gapdh* | TGTCCGTCGTGGATCTGAC | CCTGCTTCACCACCTTCTTG |

**Supplementary Table S2.**

List of 195 genes located on the 17p (17p.13) region.

| Gene |  |  |  |
| --- | --- | --- | --- |
| ABR | FBXO39 | OR3A1 | SLC43A2 |
| ACADVL | FGF11 | OR3A3 | SMG6 |
| ACAP1 | GABARAP | OVCA2 | SMTNL2 |
| ADPRM | GAS7 | P2RX1 | SMYD4 |
| AIPL1 | GEMIN4 | P2RX5-TAX1BP3 | SOX15 |
| ALOX12 | GGT6 | PAFAH1B1 | SPAG7 |
| ALOX12B | GLOD4 | PER1 | SPEM1 |
| ALOX15B | GLP2R | PFAS | SPEM2 |
| ARHGEF15 | GLTPD2 | PFN1 | SPEM3 |
| ARRB2 | GP1BA | PHF23 | SPNS2 |
| ASGR1 | GPS2 | PIK3R5 | SPNS3 |
| ASPA | GSG1L2 | PIK3R6 | SRR |
| ATP1B2 | GUCY2D | PIMREG | STX8 |
| ATP2A3 | HASPIN | PITPNA | TAX1BP3 |
| AURKB | HES7 | PITPNM3 | TEKT1 |
| BCL6B | HIC1 | PLD2 | TIMM22 |
| BHLHA9 | INCA1 | POLR2A | TLCD2 |
| BORCS6 | INPP5K | PRPF8 | TLCD3A |
| C17orf100 | ITGAE | PSMB6 | TM4SF5 |
| C17orf114 | KCNAB3 | RABEP1 | TMEM102 |
| C17orf97 | KIAA0753 | RANGRF | TMEM107 |
| C1QBP | KIF1C | RAP1GAP2 | TMEM220 |
| CAMKK1 | MED11 | RCVRN | TMEM23 |
| CAMTA2 | MED31 | RFLNB | TMEM256 |
| CCDC42 | METTL16 | RILP | TMEM88 |
| CCDC92B | MFSD6L | RNASEK | TMEM95 |
| CD68 | MINK1 | RNASEK-C17orf49 | TNFSF12-TNFSF13 |
| CFAP52 | MNT | RNF222 | TNFSF13 |
| CHD3 | MPDU1 | RNF227 | TP53 |
| CHRNB1 | MRM3 | RPA1 | TRARG1 |
| CHRNE | MYBBP1A | RPAIN | TRPV1 |
| CLEC10A | MYH1 | RTN4RL1 | TSR1 |
| CLUH | MYH2 | SAT2 | TXNDC17 |
| CNTROB | MYH3 | SCARF1 | UBE2G1 |
| CRK | MYH4 | SCGB1C2 | USP43 |
| CTC1 | MYH8 | SCIMP | USP6 |
| CTNS | NAA38 | SCO1 | VAMP2 |
| CXCL16 | NCBP3 | SENP3 | VMO1 |
| CYB5D1 | NEURL4 | SENP3-EIF4A1 | VPS53 |
| CYB5D2 | NLRP1 | SERPINF1 | WDR81 |
| DERL2 | NTN1 | SGSM2 | XAF1 |
| DHX33 | NXN | SHBG | YBX2 |
| DNAH2 | ODF4 | SHPK | YWHAE |
| DOC2B | OR1A2 | SLC13A5 | ZFP3 |
| DPH1 | OR1D2 | SLC16A13 | ZMYND15 |
| DVL2 | OR1D5 | SLC25A11 | ZNF232 |
| EFNB3 | OR1E2 | SLC25A35 | ZNF594 |
| EIF4A1 | OR1G1 | SLC2A4 | ZZEF1 |
| EMC6 | OR1R1P | SLC35G6 |  |
